# Supplementary material for: Insect Transcription Factors: A Landscape of Their Structures and Biological Functions in Drosophila and beyond
Source: Int J Mol Sci. 2018 Nov 21;19(11):3691. doi: 10.3390/ijms19113691 (PMC6274879; doi:10.3390/ijms19113691)
Supplement: Supplementary file 1 [file ijms-19-03691-s001.pdf]

**Table S1.** The structures and functions of current documented transcription factors in diverse insects.

| TFs[a] | Structures [b] | Species | Target genes | Functions | References[c] |
|---|---|---|---|---|---|
| Abd-A | A | *Bombyx mori* | *WCP4* | Cuticle protein synthesis | [1,2] |
| | | *Drosophila melanogaster* | — | NB apoptosis and quintessence | [3,4] |
| | | *Manduca sexta* | — | The expression patterns in the embryos | [5] |
| | | *Plutella xylostella* | — | Abdominal segment development | [6] |
| | | *Spodoptera litura* | — | Embryonic development | [7] |
| Abd-B | A | *Bombyx mori* | — | Mating structure | [8] |
| | | *Drosophila melanogaster* | — | Inhibition of specific NB formation in the embryonic tail region | [9] |
| AhR, ARNT | B | *Drosophila melanogaster* | *CYP6B1* | Xenobiotic response | [10] |
| | | *Aphis gossypii* | *CYP6DA2* | Gossypol tolerance | [11] |
| | | *Papilio polyxenes* | *CYP6B1v3* | Tolerance to toxic furanocoumarins | [12,13] |
| Amos | B | *Drosophila melanogaster* | — | Promoting olfactory sensory formation and inhibiting bristle formation | [14] |
| Antp | A | *Bombyx mori* | *sericin-3, fhxh4, fhxh5* | Silk synthesis | [15] |
| | | | Silk gland specific genes | The terminal differentiation of the silk gland | [16] |
| | | *Bicyclus anynana* | — | Embryonic body plan and wing patterns | [17,18] |
| | | *Drosophila melanogaster* | — | NB quintessence | [3,4] |
| | | | — | The role of YPWM motif in homeotic transformations | [19] |
| | | *Manduca sexta* | — | The expression patterns in the embryos | [5] |
| AP-4 | B | *Drosophila melanogaster* | — | Tracheal terminal branching and cell growth | [20] |
| | | *Helicoverpa armigera* | *DH-PBAN* | Diapause | [21] |
| Apt-like | A | *Bombyx mori* | The single genetic *p* | Larval pigment patterns | [22] |

| | | | | | |
|---|---|---|---|---|---|
| | | | locus | | |
| Ase | B | *Drosophila melanogaster* | — | CNS development | [23] |
| ATF-2 | B | *Drosophila melanogaster* | — | Fat metabolism | [24] |
| ATF-2, THAP | B | *Aedes aegypti* | *SCP-2* | Energy metabolism | [25] |
| ATF-3 | B | *Drosophila melanogaster* | *Or47b* | Olfactory system | [26] |
| | | | — | Metamorphosis | [27] |
| | | | — | Immune and metabolic homeostasis | [28] |
| Ato | B | *Drosophila melanogaster* | — | Chordotonal organs formation | [29] |
| | | *Bombyx mori* | — | Molecular cloning and characterization of *ato* | [30,31] |
| Awh | A | *Bombyx mori* | *fibH* | Silk protein synthesis | [16,32,33] |
| Bcd | A | *Drosophila melanogaster* | — | Embryonic axis establishment | [34,35] |
| BCFI, BCFII | C | *Bombyx mori* | chorionic gene | Regulation of chorionic gene expression | [36] |
| bHLH TFs | B | *Aedes aegypti*<br>*Anopheles gambiae*<br>*Culex quinquefasciatus* | — | Genome-wide identification | [37] |
| | | *Acyrthosiphon pisum* | — | Genome-wide identification | [38] |
| | | *Apis mellifera* | — | Genome-wide identification | [39] |
| | | *Bombyx mori* | — | Genome-wide identification | [40] |
| | | *Diaphorina citri* | — | Genome-wide identification | [41] |
| | | *Drosophila melanogaster* | — | Study of the origin and diversification of the bHLH TFs | [42] |
| | | *Harpegnathos saltator* | — | Genome-wide identification | [43] |
| | | *Leptinotarsa decemlineata* | — | Genome-wide identification | [44] |
| | | *Nasonia vitripennis* | — | Genome-wide identification | [45] |
| | | *Nilaparvata lugens* | — | Genome-wide identification | [46] |
| | | *Pediculus humanus corporis* | — | Genome-wide identification | [47] |

| | | | | | |
|---|---|---|---|---|---|
| | | *Plutella xylostella* | — | Genome-wide identification | [48] |
| | | *Tribolium castaneum* | — | Female reproduction | [49] |
| BMFA | D | *Bombyx mori* | *P25* | Silk protein synthesis | [50] |
| Bmx | B | *Drosophila melanogaster* | — | Fat metabolism | [51,52] |
| BrC | C | *Aedes aegypti* | *vitellogenin* | Vitellogenesis | [53–56] |
| | | *Bombyx mori* | — | Nuclear import of BrC | [57] |
| | | *Bombyx mori* | — | Post-transcription and -translation regulation | [58] |
| | | *Nilaparvata lugens* | — | Ovary development | [59] |
| | | *Tribolium castaneum* *Chrysopa perla* | — | Metamorphosis | [60] |
| BrC-Z1 | C | *Drosophila melanogaster* | *Lcp65A-b, Edg78E* | Pupal cuticle protein synthesis | [61] |
| | | | *phm, dib, sad* | Ecdysteroidogenesis | [62] |
| BrC-Z2 | C | *Bombyx mori* | *vitellogenin* | Reproduction | [63,64] |
| | | | *WCP10, CPG13* | Cuticle protein synthesis | [65–67] |
| BrC-Z2, BrC-Z3 | C | *Drosophila melanogaster* | Activate *E75A* and repress *E75B* under nutritional shortage | Egg chamber apoptosis | [68] |
| BrC-Z4 | C | *Drosophila melanogaster* | *npc1* | Ecdysteroidogenesis | [69] |
| | | *Bombyx mori* | *WCP5* | Cuticle protein synthesis | [70] |
| | | | *lebocin* | Immune response | [71] |
| Btd | C | *Drosophila melanogaster* | *Pros* | Nervous system development | [72] |
| Cad | A | *Acyrthosiphon pisum* | — | Early embryo development | [73] |
| | | *Drosophila melanogaster* | *ftz, spalt* | Embryonic development | [74–76] |
| | | | AMPs | Immune response and commensal-gut mutualism | [77–79] |
| | | | — | Embryo development | [9,35] |

| | | | | | |
|---|---|---|---|---|---|
| | | *Bombyx mori* | *eve* | Embryo body segmentation | [80] |
| Cato | B | *Drosophila melanogaster* | — | Sensory neuron morphology | [81] |
| CbZ | B | *Bombyx mori* | Chorion genes | Vitellogenesis | [82] |
| C/EBP | B | *Drosophila melanogaster* | — | Embryogenesis; cell migration, apical constriction and epithelial invagination during oogenesis; | [83–85] |
| | | *Bombyx mori* | Chorion genes, itself | Vitellogenesis; choriogenesis | [82,86,87] |
| | | *Anopheles gambiae* *Aedes aegypti* | *def* | Immune response | [88] |
| | | *Spodoptera litura* | *SlSCPx* | Energy metabolism | [89] |
| CncB | B | *Drosophila melanogaster* | — | Segmental diversity in the head | [90] |
| CncC | B | *Drosophila melanogaster* | *nvd, spok, dib, sad* | Ecdysteroidogenesis | [91] |
| | | | *gstD1, ref(2P, Atg8a* | Oxidative stress response | [92,93] |
| | | | *Cyp6a2, Cyp6a8, Jheh1, GstD2* and *CG6188* | Response to phenobarbital (PB; malathion and DDT resistance | [94,95] |
| | | | *Cyp6a2* | DDT resistance | [96] |
| | | | *Jheh1* and *Jheh2* (Transposable element Bari-Jheh was added in their promoters | Paraquat and malathion resistance | [97] |
| | | | — | Antioxidant response,; insecticide resistance; endocrine signaling and aging | [98,99] |
| | | *Tribolium castaneum* | CYP6BQ cluster genes | Deltamethrin resistance | [100] |
| | | | *CYP4G7, CYP4G14, GST-1, ABCA-UB, ABCAA1, ABCA-A1L* | Pyrethroid resistance | [101] |

| | | | | | |
|---|---|---|---|---|---|
| | | | and *ABCA-9B* | | |
| | | *Leptinotarsa decemlineata* | *CYP6BJa/b*, *CYP6BJ1v1*, *CYP9Z25*, and *CYP9Z29* | Imidacloprid resistance and adaptation to potato plant allelochemicals | [102] |
| | | *Aphis gossypii* | *CYP6DA2* | Gossypol tolerance | [103] |
| | | *Bombyx mori* | — | Response after phoxim and phoxim treatment | [104,105] |
| Col | B | *Drosophila melanogaster* | — | Muscle identity and shape; multidendritic neuron differentiation; head development; blood cell progenitor maintenance | [106–110] |
| Crc | B | *Drosophila melanogaster* | — | Molting and metamorphosis | [111–113] |
| CREB | B | *Drosophila melanogaster* | *period* | Circadian rhythm | [114] |
| | | | cAMP-response elements | Long-term memory | [115,116] |
| | | *Bombyx mori* | — | Diapause process induced by environmental factors; Aluminum (Al-induced neurotoxicity or neurodegeneration | [117,118] |
| | | *Cotesia glomerata* *Cotesia rubecula* | — | Long-term memory formation | [119] |
| | | *Apis mellifera* | — | Neuronal and behavioral plasticity | [120,121] |
| | | *Antheraea pernyi* | — | Identification and function of CREB | [122] |
| dCREB2 | B | *Drosophila melanogaster* | — | Circadian rhythm; formation of long-term memory | [114–116] |
| CrebA | B | *Drosophila melanogaster* | SPCGs | Embryonic SG development | [123] |
| | | | — | Secretory capacity and activity in salivary glands | [123,124] |
| | | | — | Nervous system development | [125] |

| | | | | | |
|---|---|---|---|---|---|
| CYC | B | *Nilaparvata lugens* | P450 genes | The susceptibility to imidacloprid | [126] |
| CYC, CLK | B | *Drosophila melanogaster* | *per, tim, vri, Pdp1ε, cwo* | Circadian rhythm | [127–130] |
| | | *Pyrrhocoris apterus* | $Pdp1_{iso1}$, cry2 | Adult diapause regulation | [131] |
| Cwo | B | *Drosophila melanogaster* | itself and other clock genes | Circadian rhythm | [132] |
| Da | B | *Drosophila melanogaster* | — | Photoreceptor cell determination; eye morphogenesis; cell proliferation | [133,134] |
| Dac | A | *Drosophila melanogaster* | — | Neuronal specification | [135] |
| Deaf1, lola | C | *Anopheles gambiae* | — | Immune response | [136] |
| Dei | B | *Drosophila melanogaster* | Muscle creatine kinase | Epidermal cells differentiate into muscle attachment sites | [137] |
| | | | *βPS integrin* | Wing development | [138] |
| Dichaete | D | *Drosophila melanogaster* | *commisureless, ase* | Nervous system development | [139] |
| Dif | D | *Drosophila melanogaster* | AMPs | Immune response | [140,141] |
| Dimm | B | *Drosophila melanogaster* | — | Molecular and cellular properties of all major neuroendocrine cells | [112,142–146] |
| | | | — | Protection against Gram-negative infection | [147] |
| | | *Bombyx mori* | *fibH* | Silk protein synthesis | [148,149] |
| Dfd, Lab | A | *Drosophila melanogaster* | — | Embryonic development | [150] |
| Dip3 | D | *Drosophila melanogaster* | AMPs | Immune response | [151] |
| Dl | D | *Drosophila melanogaster* | — | Embryonic axis establishment | [35,152] |
| | | *Manduca sexta* | AMPs | Immune response | [153] |
| Dm | | *Drosophila melanogaster* | *Sxl* | Sex determination | [154] |
| Dnato3 | B | *Drosophila melanogaster* | — | Nervous system development | [155] |
| Dpn | B | *Drosophila melanogaster* | *Sxl* | Sex determination | [156,157] |
| | | | *erm* | Nervous system development | [158–160] |

| | | | | | |
|---|---|---|---|---|---|
| | | | — | Mitotic activity during optic lobe development | [161] |
| | | | — | R7 specification during eye development | [162] |
| Dsx | C | *Aedes aegypti* | — | Multiple sex-specific morphological, physiological, and behavioral traits of adult females; the olfactory phenotypes | [163] |
| | | *Antheraea assama*<br>*Antheraea mylitta* | — | Female sexual differentiation | [164] |
| | | *Apis mellifera* | — | Reproductive division of labor | [165] |
| | | *Bombyx mori* | — | Sex determination; sexually dimorphic traits | [166–168] |
| | | *Cyclommatus metallifer* | — | sex-specific mandible growth | [169] |
| | | *Drosophila melanogaster* | *yolk protein* genes; *bab1/2*; *desaturase-F* | Reproduction; sexually dimorphic traits; sex determination | [170–174] |
| | | *Drosophila immigrans* species group | — | The origin and diversification of male sexual ornaments | [175] |
| | | *Nilaparvata lugens* | — | Male BPH somatic development and mating behavior | [176] |
| | | *Onthophagus taurus* | — | Sexual dimorphism | [177] |
| | | *Ostrinia scapulalis* | — | Sex determination | [178] |
| | | *Sciara* | — | Sex determination | [179] |
| | | *Spodoptera litura* | — | Testis development and the formation and function of external genitalia | [180] |
| | | *Tribolium castaneum*<br>*Onthophagus taurus* | Multiple potential Dsx targets | Sexual dimorphism | [174] |
| | | *Tribolium castaneum* | Vitellogenins, Vitellogenin receptors | Reproduction | [181] |

| | | | | | |
|---|---|---|---|---|---|
| | | *Trypoxylus dichotomus* | — | Sexual dimorphism | [182,183] |
| Dys | B | *Drosophila melanogaster* | — | Tracheal migration; dhesion and fusion; leg development | [184–187] |
| E2F4 | A | *Antheraea pernyi* | — | Immune response | [188] |
| E74 | C | *Bombyx mori* | Atg genes | Autophagy in the fat body | [189] |
| E74A | C | *Bombyx mori* | *EO* | Ecdysone degradation | [190] |
| | | *Bradysia hygida* | *BhC4-1* | Ecdysone signaling | [191] |
| E74B | C | *Aedes aegypti* | *vitellogenin* | Vitellogenesis | [192] |
| E75 | C | *Bombyx mori* | E75A, B and C repress *HR3*; E75A and E75C directly induce Halloween genes; E75B down-regulates Halloween genes | Ecdysteroidogenesis | [193] |
| | | *Aedes aegypti* | *vitellogenin* | Vitellogenesis | [53–56] |
| | | *Thermobia domestica* | — | Circadian clock; ecdysis | [194] |
| | | *Drosophila melanogaster* | *period* | Circadian clock | [195] |
| E75A | C | *Drosophila melanogaster* | *Eip63F-1* | Salivary gland development | [196] |
| | | *Manduca sexta* | *MHR3* | Ecdysone signaling | [197] |
| E75B | C | *Manduca sexta* | *ddc* | Molting | [198] |
| | | *Plodia interpunctella* | — | Differentiation of imaginal wing cells | [199] |
| E93 | C | *Bombyx mori* | — | Larval-pupal metamorphosis | [200] |
| E93, Br-C, Kr-h1 | C | *Xenos vesparum* | — | Female neoteny | [201] |
| EcR | C | *Bombyx mori* | — | Programmed cell death in the anterior silk glands; silk protein synthesis | [202,203] |

| | | | | | |
|---|---|---|---|---|---|
| | | *Bicyclus anynana* | — | Variation in wing eyespot patterns | [204] |
| | | *Drosophila melanogaster* | *Kr-h1* | Photoreceptor maturation | [205] |
| EcR, USP | C | *Drosophila melanogaster* | — | 20E signaling pathway | [206] |
| | | *Schistocerca gregaria* | — | Metamorphosis and development | [207] |
| EcR-B1 | C | *Plodia interpunctella* | — | Differentiation of imaginal wing cells | [199] |
| Emc | B | *Drosophila melanogaster* | *string* | Cell cycle and dorsal-ventral patterning of the imaginal discs | [134,208,209] |
| | | *Bombyx mori* | — | Blastokinesis | [210] |
| Ems | A | *Drosophila melanogaster* | — | Embryonic and olfactory sense organ development | [211] |
| En, Nej | A | *Drosophila melanogaster* | *dpp* | Ovarian stem cell niche | [212] |
| Erm | C | *Drosophila melanogaster* | — | Nervous system development | [160,213,214] |
| ERR, EcR, Usp | C | *Bombyx mori* | *vitellogenin* | Reproduction | [215] |
| ERR, EcR | C | *Teleogryllus emma* | — | Maintenance of the function of adult cricket testis | [216] |
| Ets | A | *Bombyx mori* | *lebocin* | Immune response | [71] |
| Eve | A | *Bombyx mori* | — | Embryo segmentation | [217] |
| Eya | D | *Drosophila melanogaster* | — | Neuronal specification | [135] |
| Fer1 | B | *Drosophila melanogaster* | odorant receptor | Odor identification | [218] |
| Fer2 | B | *Drosophila melanogaster* | — | Nervous system development; locomotor rhythms regulation | [219] |
| Fkh | A | *Drosophila melanogaster* | *Dipt, Mtk* | Immune response | [220] |
| | | | autoregulation, *CrebA, Sage* | Salivary gland development | [123,221–223] |
| | | | *reaper, hid, sens* | Maintenance of SG cells survival | [196,224,225] |

| | | | | | |
|---|---|---|---|---|---|
| | | | *PH4αSG2,PH4αSG1* | Maintenance of a uniform and patent SG lumen | [222] |
| | | | *Sgs4* | Larval SG development | [226,227] |
| | | | *Eip63F-1* | $Ca^{2+}$-signaling and secretion in SG and Malpighian tubes | [228] |
| | | | *CG6770, cabut* | Growth inhibition | [229] |
| | | | SPCGs | Secretory activity in salivary glands and epidermis | [123] |
| | | *Bombyx mori* | *fibH* | Silk protein synthesis | [230] |
| | | | *fibL, P25, sericin-1* | Silk protein synthesis | [231–233] |
| | | *Manduca sexta* | *moricin, lysozyme, defensin-1, defensin-3, attacin-2* | Immune response | [234] |
| FoxA | A | *Helicoverpa armigera* | *DH-PBAN* | Diapause | [235] |
| | | *Helicoverpa armigera Spodoptera litura* | *ABCC2, ABCC3* | Cry1Ac toxin resistance | [236] |
| FoxL1 | A | *Drosophila melanogaster* | *Sema2a* | SG positioning in embryos | [237] |
| FoxL2 | A | *Nilaparvata lugens* | *Fcp3C* | Choriogenesis | [238] |
| FOXO | A | *Drosophila melanogaster* | *lipase 4, brummer* | Lipid metabolism | [239,240] |
| | | | *Drs* | Immune response | [241] |
| | | | — | Ecdysone biosynthesis | [242] |
| | | *Bombyx mori* | *brummer, acid lipase-1* | Lipolysis | [243] |
| | | | *JHE, JHEH, JHDK* | JH degradation | [244] |
| | | *Glossina morsitans* | — | Lipolysis suppression | [245] |
| βFtz-F1 | C | *Blattella germanica* | — | JH biosynthesis | [246] |
| | | *Drosophila melanogaster* | — | JH signaling | [247] |

| | | | | | |
|---|---|---|---|---|---|
| | | | *phm, dib, sad* | Ecdysteroidogenesis | [248,249] |
| | | | *EDG84A, EDG78E* | Pupal cuticle protein synthesis | [250–252] |
| | | | *MMP2* | Fat body remodeling | [253] |
| | | *Bombyx mori* | *BmWCP5, BmWCP4, CPR55* | Cuticle protein synthesis | [66,67,70,254] |
| | | | *BmACP-6.7* | Formation of adult epidermis | [255] |
| | | | Atg genes | Autophagy in the fat body | [189] |
| | | | *fibH* | Silk protein synthesis | [256] |
| | | *Aedes aegypti* | *vitellogenin* | Vitellogenesis | [257] |
| FTZ | A | *Drosophila melanogaster* | *slp1, wg, en* | Embryonic axis establishment | [258,259] |
| GATAβ | C | *Bombyx mori* | late chorion genes | Choriogenesis | [36,260,261] |
| GATA-1 | C | *Manduca sexta* | *moricin* | Immune response | [262] |
| Gce | B | *Drosophila melanogaster* | — | A cofactor of Met | [263] |
| Gt | B | *Drosophila melanogaster* | *kr, hb, eve* | Embryonic development | [264] |
| Hand | B | *Drosophila melanogaster* | — | Cardiogenesis; hematopoiesis; wing heart and muscle formation | [265–268] |
| H, Gro | B | *Aedes aegypti* | 15% of Met-repressed genes | Reproduction | [269] |
| Hb | A | *Bombyx mori* | — | Embryo development | [270] |
| | | *Drosophila melanogaster* | — | NB cell fate decision | [271] |
| | | | *rhomboid, Star, CBP, Fasciclin2, sprout* | Retinal glia cell development and blood-brain barrier integrity | [272] |
| HNF4 | C | *Aedes aegypti* | — | Lipid metabolism | [273] |
| bHLH54F | B | *Drosophila melanogaster* | — | Expressed in the precursor of longitudinal visceral muscle | [274] |
| Hinge 1/2/3 | D | *Drosophila melanogaster* | — | Wing hinge formation | [275] |

| | | | | | |
|---|---|---|---|---|---|
| DHR3 | C | *Drosophila melanogaster* | *EDG84A* | Pupal cuticle protein synthesis | [250,252] |
| | | | *EcR, E74B, βFTZ-1* | Metamorphosis | [276] |
| | | | *phm, dib, sad* | Ecdysteroidogenesis | [249] |
| HR3 | C | *Aedes aegypti* | *SCP-2* | Energy metabolism | [257] |
| | | | *vitellogenin* | Vitellogenesis | [53–56] |
| | | *Bombyx mori* | Atg genes | Autophagy in the fat body | [189] |
| | | *Manduca sexta* | *MHR3* | Ecdysone signaling | [197] |
| | | *Plodia interpunctella* | — | Differentiation of imaginal wing cells | [199] |
| | | *Thermobia domestica* | — | Circadian clock; ecdysis | [194] |
| DHR4 | C | *Drosophila melanogaster* | *Cyp6t3* | Repress ecdysteroidogenesis | [277] |
| HR4 | C | *Manduca sexta* | *ddc* | Molting | [198] |
| HR38 | C | *Bombyx mori* | — | The effect of PTTH on HR38 expression | [278] |
| DHR39 | C | *Drosophila melanogaster* | *EDG84A* | Pupal cuticle protein synthesis | [250,252] |
| DHR96 | C | *Drosophila melanogaster* | Many PB-regulated genes | PB tolerance and DDT resistance | [279] |
| HSF | A | *Drosophila melanogaster* | Heat shock genes | Heat stress | [280,281] |
| | | *Bombyx mori* | — | Activation of *Hsp70a* and *Samui* under 5 ℃ -incubation | [282] |
| Hth | A | *Drosophila melanogaster* | — | Development of the wing imaginal disc | [283,284] |
| ILF | C | *Bombyx mori* | *POUM2* | Metamorphosis | [285] |
| Keap1 | D | *Bombyx mori* | — | Response after phoxim and phoxim treatment | [104,105] |
| Kni | C | *Drosophila melanogaster* | *Phm, Dib* | Ecdysteroidogenesis | [286] |
| Kr | C | *Drosophila melanogaster* | *Rh5, Rh6* | Photoreceptor neuron differentiation | [287] |
| Kr-h1 | C | *Bombyx mori* | *Br-C, E93* | Metamorphosis | [288,289] |
| Kr-h1 | C | *Drosophila melanogaster* | Steroidogenic enzymes | Ecdysone biosynthesis; metabolic homeostasis; metamorphosis | [240,290,291] |

| | | | | | |
|---|---|---|---|---|---|
| | | *Locusta migratoria* | *vitellogenin* | Vitellogenesis and oocyte maturation | [292] |
| | | *Nilaparvata lugens* | — | Ovary development | [59] |
| | | *Tribolium castaneum*<br>*Blattella germanica* | *E93* | Metamorphosis | [290,293] |
| | | *Pyrrhocoris apterus* | *vitellogenin* | Vitellogenesis | [294] |
| LL3 | D | *Anopheles gambiae* | *SPRN6* | Anti-Plasmodium immunity | [295] |
| *l'sc* | B | *Drosophila melanogaster* | — | Muscle progenitors | [296] |
| Lolal | D | *Drosophila melanogaster* | *crumbs, Moesin* | SG and trache elongation | [297] |
| Islet, Lim3 | A | *Drosophila melanogaster* | — | Motoneuron identity | [298] |
| Max | B | *Drosophila melanogaster* | — | Regulation of Myc activity | [299] |
| MBF2 | D | *Bombyx mori* | *fibH* | Silk protein synthesis | [149] |
| Met | B | *Bombyx mori* | *Kr-h1* | Metamorphosis | [300,301] |
| | | *Cimex lectularius* | *vitellogenin* | Vitellogenesis and ovigenesis | [302] |
| | | *Drosophila melanogaster* | *Dronc, Drice* | Programmed cell death | [303] |
| | | *Glossina morsitans* | — | Lipolysis suppression | [245] |
| | | *Leptinotarsa decemlineata* | — | Growth and development | [304,305] |
| | | *Locusta migratoria* | *vitellogenin* | Vitellogenesis and oocyte maturation | [292] |
| | | | *Mcm4, Mcm7* | Polyploidy and vitellogenesis | [306] |
| | | | *Cdc6, Grp78-2* | Fat body cell homeostasis, polyploidy, vitellogenesis and oogenesis | [307,308] |
| | | *Pyrrhocoris apterus* | *vitellogenin* | Vitellogenesis | [294] |
| | | | Accessory gland proteins and hexamerins | Male reproduction | [309] |
| | | | *Pdp1$_{iso1}$, cry2* | Adult diapause regulation | [131] |
| | | *Tribolium castaneum* | *Kr-h1, Br-C* | Metamorphosis | [60,290,310–314] |

| | | | | | |
|---|---|---|---|---|---|
| Met, SRC | B | *Aedes aegypti* | *Kr-h1;RRS1*; six ribosomal protein; *ET* | Reproduction | [315–318] |
| Met, CYC, SRC | B | *Aedes aegypti* | *Kr-h1, Hairy* | Light-dependent circadian regulation | [319] |
| MIC | D | *Bombyx mori* | *sericin-1* | Silk protein synthesis | [320–322] |
| Mid | D | *Drosophila melanogaster* | *wingless, hedgehog* | Wing disc development | [323] |
| | | | *gooseberry* | Neuroblast development | [324] |
| Mitf | B | *Drosophila melanogaster* | — | Eye development | [325] |
| | | | V-ATPase | Lysosomal-autophagy pathway | [326] |
| Mld | C | *Drosophila melanogaste* | *spok* | Ecdysteroidogenesis | [286,327–329] |
| Mlx, Mondo | B | *Drosophila melanogaster* | *Cabut, Aldh-III* | Dietary sugar tolerance | [330] |
| Mnt | B | *Drosophila melanogaster* | — | Body size regulation | [331] |
| dMyd | B | *Drosophila melanogaster* | — | A transiently expressed muscle marker | [332] |
| Nej, Tgo | B | *Drosophila melanogaster* | — | Peripheral glial differentiation | [333] |
| Nkx-2.5-like, transcription factor 3-like | A | *Apis mellifera* | Unknown | Fipronil resistance | [334] |
| Odd | C | *Drosophila melanogaster* | — | Embryonic axis establishment | [335] |
| | | *Bombyx mori* | — | Embryo segmentation | [217] |
| Oli | B | *Drosophila melanogaster* | — | Nervous system development; larval and adult locomotion; neuronal remodeling | [336,337] |
| Otd | A | *Drosophila melanogaster* | *kr-h1* | Photoreceptor maturation | [205] |
| | | *Bombyx mori* | *eve* | Embryo body segmentation | [80] |
| | | *Onthophagus binodis* *Digitonthophagus gazella* *Liatongus militaris* | — | Development of functional ectopic compound eyes | [338] |

| | | | | | |
|---|---|---|---|---|---|
| Pb | A | *Drosophila melanogaster* | — | Embryonic axis establishment | [339] |
| Pdm1, Pdm2 | A | *Drosophila melanogaster* | *CecA1* | Immune response | [340] |
| Pdp1 | B | *Drosophila melanogaster* | — | Regulation of somatic muscle gene expression | [341] |
| Pdp1ε | B | *Drosophila melanogaster* | — | Circadian rhythm | [130] |
| Pdp1γ | B | *Drosophila melanogaster* | — | Pphysiological and drug-influenced lipid regulation | [342] |
| Pitx | A | *Bombyx mori* | *DH-PBAN, PTTH* | Neuroendocrine system activation | [343,344] |
| PntP1 | A | *Drosophila melanogaster* | *Ase, erm, Pros* | Nervous system development | [345,346] |
| POU-M1 | A | *Bombyx mori* | *sericin-1* | Silk protein synthesis | [321,347] |
| Prd | A | *Drosophila melanogaster* | *EcR* | The function of male accessory glands and fertility | [348] |
| Prey2 | D | *Helicoverpa armigera* | *CYP6B6* | Response to plant secondary toxicant 2-tridecanone | [349] |
| Vri | B | *Drosophila melanogaster* | — | A dominant enhancer of Dpp signaling in embryonic D-V patterning and wings; cardiac aging; hair and cell growth; embryonic tracheal development; circadian clock | [350–353] |
| | | *Spodoptera frugiperda* | — | Circadian rhythm regulation | [354] |
| Vvl | A | *Drosophila melanogaster* | *CecA1, DiptA, AttA, AttB, Drs* | Immune response | [79,340] |
| | | | *Phm, Dib* | Ecdysteroidogenesis | [286] |
| | | | — | Motoneuron identity | [298] |
| | | *Bombyx mori* | *vitellogenin* | Reproduction | [64] |
| | | | *fibH* | Silk synthesis | [355] |
| | | | *Phm* | Ecdysteroidogenesis | [356] |

| | | | | | |
|---|---|---|---|---|---|
| | | | *DH-PBAN* | Diapause | [357] |
| | | | *BmWCP4* | Cuticle protein synthesis | [1,2] |
| | | *Tribolium castaneum* | *jhamt3, HR3,Phm, Spo* | JH biosynthesis and ecdysteroidogenesis | [358] |
| | | *Helicoverpa armigera* | *DH-PBAN, PTEN* | Diapause | [359,360] |
| Rel | D | *Drosophila melanogaster* | — | Immune response | [141,361,362] |
| | | *Helicoverpa armigera* | *cathepsin L* | Fat body dissociation | [363] |
| Rel2 | D | *Manduca sexta* | AMPs | Immune response | [153] |
| REL2 | D | *Anopheles gambiae* | AMPs | Immune response | [364] |
| REL1-A, REL1-B | D | *Aedes aegypti* | *Dipt, Drs,* | Antifungal immune responses | [365] |
| REL2 | D | *Aedes aegypti* | *Defensins A, C, D Cec A, Cec N* | Defense against Gram-positive, Gram-negative bacteria and *Plasmodium gallinaceum* | [366–368] |
| Rel2 | D | *Culex quinquefasciatus* | *Vago* | Anti-viral responses | [369] |
| RelA, RelB | D | *Bombyx mori* | *Lebocin4, Attacin* | Immune response | [370] |
| Rel1, Rel2 | D | *Bombyx mori* | *Cecropin B1, Attacin, Lebocin 4* | Immune response | [371] |
| RelA | D | *Allomyrina dichotoma* | *Coleoptericin A* | Immune response | [372] |
| Rib | A | *Drosophila melanogaster* | *crumbs, Moesin,* | SG and trache elongation | [297] |
| | | | Autoregulation | Embryonic SG morphogenesis | [373] |
| Run | D | *Drosophila melanogaster* | *Slx* | Sex determination | [374] |
| | | | *engrailed, slp1* | Embryonic development | [258,259] |
| | | *Bombyx mori* | — | Embryo segmentation | [217] |
| Sage | B | *Bombyx mori* | *fibH* | Silk protein synthesis | [148,230] |
| | | *Drosophila melanogaster* | Sage target genes | Salivary gland development | [223] |
| | | | *PH4αSG2,PH4αSG1* | Maintenance of a uniform and patent SG | [222] |

| | | | | | |
|---|---|---|---|---|---|
| | | | | lumen | |
| Sal | C | *Pieris rapae* | — | Wing pattern development | [375] |
| Sal, E75 | C | *Papilio Xuthus* | — | Larval eyespot markings | [376] |
| Sc (Sis-B) | B | *Drosophila melanogaste* | *Sxl* | Sex determination | [377] |
| Scr | C | *Drosophila melanogaster* | *Fkh, CrebA, Sage, Hkb* | Salivary gland specification | [378–381] |
| | | *Bombyx mori* | — | Development of the embryonic silk gland | [382] |
| Séan, Ouib, Mld | C | *Drosophila melanogaster* | *nvd, spok* | Ecdysteroidogenesis | [329] |
| Sens | C | *Drosophila melanogaster* | *reaper, hid* | Maintenance of salivary gland cells survival | [225] |
| SGF 1/2/3/4 | D | *Bombyx mori* | — | Silk protein synthesis | [231,383] |
| Sim, Tgo | B | *Drosophila melanogaster* | — | CNS midline cell development | [384,385] |
| Sima, Tgo | B | *Drosophila melanogaster* | — | Normal development and hypoxia response | [386,387] |
| Sis-A | B | *Drosophila melanogaster* | *Sxl* | Sex determination | [388] |
| | | | — | Midgut formation; Brahma complex recruitment | [389] [390] |
| slp1 | A | *Drosophila melanogaster* | — | Embryonic axis establishment | [259] |
| Sna, Esg, Wor | C | *Drosophila melanogaster* | — | Asymmetric cell division of embryonic NBs | [391–393] |
| Nine Sox genes | D | *Bombyx mori* | — | Identification and characterization of Sox genes | [394] |
| SRAM | D | *Sarcophaga peregrina* | *lectin* | Immune response | [395] |
| dSREBP | B | *Drosophila melanogaster* | — | Lipid synthesis | [396] |

| | | | | | |
|---|---|---|---|---|---|
| | | | — | Nervous system development | [397] |
| SRC | B | *Cimex lectularius* | — | Reproduction | [398] |
| | | *Aedes aegypti* | — | Reproduction | [315,316,318] |
| | | *Locusta migratoria* | — | Reproduction | [292,306–308] |
| | | *Pyrrhocoris apterus* | — | Reproduction | [294,309] |
| | | *Bombyx mori* | — | Metamorphosis | [300,301] |
| | | *Tribolium castaneum* | — | Metamorphosis | [313,314] |
| Ss | B | *Drosophila melanogaster* | — | Nervous system development | [186] |
| | | | — | Distal antenna formation | [399] |
| STAT | D | *Drosophila melanogaster* | *Raf* | Immune response | [400] |
| STAT-A | D | *Anopheles gambiae* | *NOS, SOCS* | Immune response | [401] |
| STAT-B | D | *Anopheles gambiae* | *STAT-A* | Immune response | [401] |
| Svp | C | *Blattella germanica* | — | JH biosynthesis | [246] |
| | | *Drosophila melanogaster* | — | CNS and photoreceptor cell development | [402–404] |
| Tgo | B | *Drosophila melanogaster* | — | Formation of CNS midline antennal and tarsal development peripheral glial cell migration | [405–408] |
| Tim | B | *Laodelphax striatellus* | — | The circadian behavioral rhythms | [409] |
| Tll | C | *Drosophila melanogaster* | — | Neural and embryonic development | [206,410,411] |
| Toy | A | *Drosophila melanogaster* | *ey* | Eye development | [412] |
| Trh, Tgo | C | *Drosophila melanogaster* | *jing* | Tracheal development | [407] |
| Twi | B | *Drosophila melanogaster* | — | Cell fate decisions | [413] |
| Tsh | C | *Drosophila melanogaster* | — | Wing imaginal disc development | [283,284] |
| Ubx | A | *Bicyclus anynana* | — | Embryonic body plan and wing patterns | [17,18] |
| | | *Drosophila melanogaster* | *dad* | The differentiation of the gastric stem cell lineage | [414] |

| | | | | | |
|---|---|---|---|---|---|
| | | *Manduca sexta* | — | The expression patterns in the embryos | [5] |
| | | *Bombyx mori* | — | The abdominal appendage specification | [415] |
| USF | B | *Drosophila melanogaster* | *Stellate* | Male fertility | [416] |
| Woc | C | *Drosophila melanogaster* | — | Ecdysone biosynthesis | [417] |
| Zld | C | *Drosophila melanogaster* | *Sxl* | Sex determination | [418] |
| | | | *eve* | Embryonic development | [419] |
| Zfh1, Zfh2 | C | *Drosophila melanogaster* | — | Embryonic development and immune regulation | [420,421] |

[a] Abbreviations of TFs in this table. **Abd-A/B**: Abdominal-A/B; **AhR**: Aryl hydrocarbon receptor; **ARNT**: Aryl hydrocarbon receptor nuclear translocator; **Antp**: Antennapedia; **Apt-like**: Apontic-like; **Ase**: Asense; **ATF-2/3**: Activating transcription factor 2/3; **Ato**: Atonal; **Bcd**: Bicoid; **Bmx**: Bigmax; **BrC**: Broad-Complex; **BrC-Z1/2/3/4**: Broad complex isoform 1/2/3/4; **Btd**: Buttonhead; **Cad**: Caudal; **Cato**: Cousin of atonal; **C/EBP**: CCAAT-enhancer-binding protein; **Cnc B/C**: Cap 'n' collar B/C; **Col**: Collier; **Crc**: Cryptocephal; **CREB**: cAMP response element binding protein; **Cyc**: Cycle; **Clk**: Clock; **Cwo**: Clockwork orange; **Da**: Daughterless; **Dac**: Dachshund; **Dei**: Delilah; **Deaf-1**: Deformed epidermal autoregulatory factor-1; **Dfd**: Deformed; **Dif**: Dorsal-related immunity factor; **Dimm**: Dimmed; **Dip3**: Dorsal interacting protein 3; **Dl**: Dorsal; **Dm**: Diminutive; **Dpn**: Deadpan; **Dys**: Dysfusion; **E74/75**: Ecdysone-induced protein 74/75; **EcR**: Ecdysone receptor; **Emc**: Extramacrochaetae; **Ems**: Empty spiracles; **En**: Engrailed; **ERR**: Oestrogen-related receptor; **Esg**: Escargot; **Eve**: Even-skipped; **Eya**: Eyes absent; **Fkh**: Forkhead; **FoxA**: Forkhead box, sub-group A; **FoxL1/2**: Forkhead box transcription factor L1/2; **FOXO**: Forkhead box, sub-group O; β**Ftz-F1**: β-fushi tarazu transcription factor 1; **Ftz**: Fushi tarazu; **Gce**: Germ-cell expressed; **Gro**: Groucho; **Gt**: Giant; **H**: Hairy; **Hb**: Hunchback; **HNF4**: Hepatocyte nuclear factor 4; **DHR3/4/39/96**: *Drosophila* Hormone Receptor 3/4/39/96; **HR3/4/38**: Hormone Receptor 3/4/38; **HSF**: Heat shock factor; **Hth**: Homothorax **Kni**: Knirps; **Kr**: Krüppel; **Kr-h1**: Krüppel homolog 1; **Lab**: Labial; **LL3**: LITAF-like 3; **l'sc**: lethal of scute; **Lolal**: Lola Like; **MBF2**: Multiprotein bridging factor 2; **Met**: Methoprene-tolerant; **Nej**: Nejire; **Odd**: Odd-skipped; **Oli**: Olig family; **Otd**: Orthodenticle; **Pdp1:** PAR-domain protein 1; **PntP1**: Pointed P1; **Prd**: Paired; **Vri**: Vrille; **USF**: Upstream stimulatory factor; **Vvl**: Ventral veins lacking; **Rel**: Relish; **Pb**: Proboscidea; **Rib**: Ribbon; **Run**: Runt; **Sal**: Spalt; **Sc**: Scute; **Scr**: Sex combs reduced; **Séan**: Séance; **Sens**: Senseless; **Sim**: Single-minded; **Sima**: Similar; **Sis-A/B**: Sisterless A/B; **Slp1**: Sloppy-paired 1; **Sna**: Snail; **SRAM**: Sarcophaga-derived Rel/Ankyrin molecule; **SRC**: Steroid receptor coactivator; **Ss**: Spineless; **STAT**: Signal transducers and activators of transcription; **Svp**: Seven-up; **Tgo**: Tango; **Tim**: Timeless; **Tll**: Tailess; **Toy**: Twin of eyeless; **Trh**: Trachealess; **Twi**: Twist; **Tsh**: Teashirt; **Ubx**: Ultrabithorax; **Wor**: Worniu; **Woc**: Without children; **Zld**: Zelda; **Zfh1**: Zn finger homeodomain 1.

[b] The structures of TF superclasses: A represents the helix-turn-helix domain factor; B represents the basic domain factor; C represents the zinc finger domain factor; D represents other DNA-binding domain factor.

[c] We apologize in advance to those whose work could not be adequately discussed and cited in the main body due to space limitations.

## References


1. Deng, H.; Zhang, J.; Li, Y.; Zheng, S.; Liu, L.; Huang, L.; Xu, W.H.; Palli, S.R.; Feng, Q. Homeodomain POU and Abd-A proteins regulate the transcription of pupal genes during metamorphosis of the silkworm, *Bombyx mori*. *Proc. Natl. Acad. Sci. U. S. A.* **2012**, *109*, 12598–12603.
2. He, Y.; Deng, H.; Hu, Q.; Zhu, Z.; Liu, L.; Zheng, S.; Song, Q.; Feng, Q. Identification of the binding domains and key amino acids for the interaction of the transcription factors BmPOUM2 and BmAbd-A in *Bombyx mori*. *Insect Biochem. Mol. Biol.* **2017**, *81*, 41–50.
3. Bello, B.C.; Hirth, F.; Gould, A.P. A pulse of the *Drosophila* Hox protein Abdominal-A schedules the end of neural proliferation via neuroblast apoptosis. *Neuron* **2003**, *37*, 209–219.
4. Tsuji, T.; Hasegawa, E.; Isshiki, T. Neuroblast entry into quiescence is regulated intrinsically by the combined action of spatial Hox proteins and temporal identity factors. *Development* **2008**, *135*, 3859–3869.
5. Zheng, Z.; Khoo, A.; Fambrough, D., Jr.; Garza, L.; Booker, R. Homeotic gene expression in the wild-type and a homeotic mutant of the moth *Manduca sexta*. *Dev. Genes Evol.* **1999**, *209*, 460–472.
6. Huang, Y.; Chen, Y.; Zeng, B.; Wang, Y.; James, A.A.; Gurr, G.M.; Yang, G.; Lin, X.; Huang, Y.; You, M. CRISPR/Cas9 mediated knockout of the *abdominal-A* homeotic gene in the global pest, diamondback moth (*Plutella xylostella*). *Insect Biochem. Mol. Biol.* **2016**, *75*, 98–106.
7. Bi, H.L.; Xu, J.; Tan, A.J.; Huang, Y.P. CRISPR/Cas9-mediated targeted gene mutagenesis in *Spodoptera litura*. *Insect Science* **2016**, *23*, 469–477.
8. Duan, J.; Xu, H.; Ma, S.; Guo, H.; Wang, F.; Zhang, L.; Zha, X.; Zhao, P.; Xia, Q. Ectopic expression of the male BmDSX affects formation of the chitin plate in female *Bombyx mori*. *Mol. Reprod. Dev.* **2014**, *81*, 240–247.
9. Birkholz, O.; Vef, O.; Rogulja-Ortmann, A.; Berger, C.; Technau, G.M. *Abdominal-B* and *caudal* inhibit the formation of specific neuroblasts in the *Drosophila* tail region. *Development* **2013**, *140*, 3552–3564.
10. Brown, R.P.; McDonnell, C.M.; Berenbaum, M.R.; Schuler, M.A. Regulation of an insect cytochrome P450 monooxygenase gene (*CYP6B1*) by aryl hydrocarbon and xanthotoxin response cascades. *Gene* **2005**, *358*, 39–52.
11. Peng, T.; Chen, X.; Pan, Y.; Zheng, Z.; Wei, X.; Xi, J.; Zhang, J.; Gao, X.; Shang, Q. Transcription factor *aryl hydrocarbon receptor/aryl hydrocarbon receptor nuclear translocator* is involved in regulation of the xenobiotic tolerance-related cytochrome P450 *CYP6DA2* in *Aphis gossypii* Glover. *Insect Mol. Biol.* **2017**, *26*, 485–495.
12. Petersen, R.A.; Niamsup, H.; Berenbaum, M.R.; Schuler, M.A. Transcriptional response elements in the promoter of *CYP6B1*, an insect P450 gene regulated by plant chemicals. *Biochim. Biophys. Acta* **2003**, *1619*, 269–282.
13. Petersen Brown, R.; Berenbaum, M.R.; Schuler, M.A. Transcription of a lepidopteran cytochrome P450 promoter is modulated by multiple elements in its 5' UTR and repressed by 20-hydroxyecdysone. *Insect Mol. Biol.* **2004**, *13*, 337–347.
14. zur Lage, P.I.; Prentice, D.R.; Holohan, E.E.; Jarman, A.P. The *Drosophila* proneural gene *amos* promotes olfactory sensillum formation and suppresses bristle formation. *Development* **2003**, *130*, 4683–4693.
15. Tsubota, T.; Tomita, S.; Uchino, K.; Kimoto, M.; Takiya, S.; Kajiwara, H.; Yamazaki, T.; Sezutsu, H. A Hox Gene, Antennapedia, regulates expression of multiple major silk protein genes in the silkworm *Bombyx mori*. *J. Biol. Chem.* **2016**, *291*, 7087–7096.
16. Takiya, S.; Tsubota, T.; Kimoto, M. Regulation of silk genes by Hox and homeodomain proteins in the terminal differentiated silk gland of the silkworm *Bombyx mori*. *J. Dev. Biol.* **2016**, *4*,
17. Saenko, S.V.; Marialva, M.S.; Beldade, P. Involvement of the conserved Hox gene Antennapedia in the development and evolution of a novel trait. *EvoDevo* **2011**, *2*, 9.
18. Tong, X.; Hrycaj, S.; Podlaha, O.; Popadic, A.; Monteiro, A. Over-expression of Ultrabithorax alters embryonic body plan and wing patterns in the butterfly *Bicyclus anynana*. *Dev. Biol.* **2014**, *394*, 357–366.
19. Prince, F.; Katsuyama, T.; Oshima, Y.; Plaza, S.; Resendez-Perez, D.; Berry, M.; Kurata, S.; Gehring,

W.J. The YPWM motif links Antennapedia to the basal transcriptional machinery. *Development* **2008**, *135*, 1669–1679.
20. Wong, M.M.; Liu, M.F.; Chiu, S.K. Cropped, *Drosophila* transcription factor AP-4, controls tracheal terminal branching and cell growth. *BMC Dev. Biol.* **2015**, *15*, 20.
21. Hu, C.H.; Hong, B.; Xu, W.H. Identification of an E-box DNA binding protein, activated protein 4, and its function in regulating the expression of the gene encoding diapause hormone and pheromone biosynthesis-activating neuropeptide in *Helicoverpa armigera*. *Insect Mol. Biol.* **2010**, *19*, 243–252.
22. Yoda, S.; Yamaguchi, J.; Mita, K.; Yamamoto, K.; Banno, Y.; Ando, T.; Daimon, T.; Fujiwara, H. The transcription factor Apontic-like controls diverse colouration pattern in caterpillars. *Nat. Commun.* **2014**, *5*, 4936.
23. González, F.; Romani, S.; Cubas, P.; Modolell, J.; Campuzano, S. Molecular analysis of the *asense* gene, a member of the *achaete-scute* complex of *Drosophila melanogaster*, and its novel role in optic lobe development. *EMBO J.* **1989**, *8*, 3553–3562.
24. Okamura, T.; Shimizu, H.; Nagao, T.; Ueda, R.; Ishii, S. ATF-2 regulates fat metabolism in *Drosophila*. *Mol. Biol. Cell* **2007**, *18*, 1519–1529.
25. Peng, R.; Fu, Q.; Hong, H.; Schwaegler, T.; Lan, Q. THAP and ATF-2 regulated sterol carrier protein-2 promoter activities in the larval midgut of the yellow fever mosquito, *Aedes aegypti*. *PLoS One* **2012**, *7*, e46948.
26. Bhat, S.; Jones, W.D. An accelerated miRNA-based screen implicates Atf-3 in *Drosophila* odorant receptor expression. *Sci. Rep.* **2016**, *6*, 20109.
27. Sekyrova, P.; Bohmann, D.; Jindra, M.; Uhlirova, M. Interaction between *Drosophila* bZIP proteins Atf3 and Jun prevents replacement of epithelial cells during metamorphosis. *Development* **2010**, *137*, 141–150.
28. Rynes, J.; Donohoe, C.D.; Frommolt, P.; Brodesser, S.; Jindra, M.; Uhlirova, M. Activating transcription factor 3 regulates immune and metabolic homeostasis. *Mol. Cell Biol.* **2012**, *32*, 3949–3962.
29. Jarman, A.P.; Grau, Y.; Jan, L.Y.; Jan, Y.N. *atonal* is a proneural gene that directs chordotonal organ formation in the *Drosophila* peripheral nervous system. *Cell* **1993**, *73*, 1307–1321.
30. Yu, L.; Zhou, Q.; Zhang, C.; Pignoni, F. Identification of *Bombyx* atonal and functional comparison with the *Drosophila* atonal proneural factor in the developing fly eye. *Genesis* **2012**, *50*, 393–403.
31. Hu, P.; Feng, F.; Xia, H.; Chen, L.; Yao, Q.; Chen, K. Molecular cloning and characterization of a *Bombyx mori* gene encoding the transcription factor Atonal. *Z. Naturforsch C* **2014**, *69*, 155–164.
32. Ohno, K.; Sawada, J.; Takiya, S.; Kimoto, M.; Matsumoto, A.; Tsubota, T.; Uchino, K.; Hui, C.C.; Sezutsu, H.; Handa, H., et al. Silk gland factor-2, involved in fibroin gene transcription, consists of LIM homeodomain, LIM-interacting, and single-stranded DNA-binding proteins. *J. Biol. Chem.* **2013**, *288*, 31581–31591.
33. Kimoto, M.; Tsubota, T.; Uchino, K.; Sezutsu, H.; Takiya, S. LIM-homeodomain transcription factor Awh is a key component activating all three fibroin genes, *fibH*, *fib*L and *fhx*, in the silk gland of the silkworm, *Bombyx mori*. *Insect Biochem. Mol. Biol.* **2015**, *56*, 29–35.
34. Nasiadka, A.; Dietrich, B.H.; Krause, H.M. Anterior-posterior patterning in the *Drosophila* embryo. In *Advances in Developmental Biology and Biochemistry*, DePamphilis, M.L., Ed. Elsevier: Amsterdam, 2002; Vol. 12, pp. 155–204.
35. Gilbert, S.F. The genetics of axis specification in *Drosophila*. In *Developmental Biology (7th edn)*, Gilbert, S.F., Ed. Sinauer Associates: Sunderland, 2003; pp. 263–304.
36. Skeiky, Y.A.; Iatrou, K. Synergistic interactions of silkmoth chorion promoter-binding factors. *Mol. Cell. Biol.* **1991**, *11*, 1954–1964.
37. Zhang, D.B.; Wang, Y.; Liu, A.K.; Wang, X.H.; Dang, C.W.; Yao, Q.; Chen, K.P. Phylogenetic analyses of vector mosquito basic helix-loop-helix transcription factors. *Insect Mol. Biol.* **2013**, *22*, 608–621.
38. Dang, C.W.; Wang, Y.; Chen, K.P.; Yao, Q.; Zhang, D.B.; Guo, M. The basic helix-loop-helix transcription factor family in the pea aphid, *Acyrthosiphon pisum*. *J. Insect Sci.* **2011**, *11*, 84.
39. Wang, Y.; Chen, K.; Yao, Q.; Wang, W.; Zhu, Z. The basic helix-loop-helix transcription factor

family in the honey bee, *Apis mellifera*. *J. Insect Sci.* **2008**, *8*, 1–12.
40. Wang, Y.; Chen, K.; Yao, Q.; Wang, W.; Zhi, Z. The basic helix-loop-helix transcription factor family in *Bombyx mori*. *Dev. Genes Evol.* **2007**, *217*, 715–723.
41. Peng, Y.; Wang, Y.; Tao, X.F.; Zeng, Z.; Johnson, N.A.N.; Yao, Q.; Chen, K.P. A genome-wide survey and analysis of basic helix-loop-helix genes in the Asian citrus psyllid, *Diaphorina citri* (Hemiptera: Psyllidae). *J. Asia Pac. Entomol.* **2017**, *20*, 821–829.
42. Simionato, E.; Ledent, V.; Richards, G.; Thomas-Chollier, M.; Kerner, P.; Coornaert, D.; Degnan, B.M.; Vervoort, M. Origin and diversification of the basic helix-loop-helix gene family in metazoans: insights from comparative genomics. *BMC Evol. Biol.* **2007**, *7*, 33.
43. Liu, A.; Wang, Y.; Dang, C.; Zhang, D.; Song, H.; Yao, Q.; Chen, K. A genome-wide identification and analysis of the basic helix-loop-helix transcription factors in the ponerine ant, *Harpegnathos saltator*. *BMC Evol. Biol.* **2012**, *12*, 165.
44. Fu, K.Y.; Meng, Q.W.; Lü, F.G.; Guo, W.C.; Ahmat, T.; Li, G.Q. The basic helix–loop–helix transcription factors in the Colorado potato beetle *Leptinotarsa decemlineata*. *J. Asia Pac. Entomol.* **2015**, *18*, 197–203.
45. Liu, X.T.; Wang, Y.; Wang, X.H.; Tao, X.F.; Yao, Q.; Chen, K.P. A genome-wide identification and classification of basic helix-loop-helix genes in the jewel wasp, *Nasonia vitripennis* (Hymenoptera: Pteromalidae). *Genome* **2014**, *57*, 525–536.
46. Wan, P.J.; Yuan, S.Y.; Wang, W.X.; Chen, X.; Lai, F.X.; Fu, Q. A genome-wide identification and analysis of the basic helix-loop-helix transcription factors in brown planthopper, *Nilaparvata lugens*. *Genes* **2016**, *7*, 100.
47. Wang, X.H.; Wang, Y.; Zhang, D.B.; Liu, A.K.; Yao, Q.; Chen, K.P. A genome-wide identification of basic helix-loop-helix motifs in *Pediculus humanus corporis* (Phthiraptera: Pediculidae). *J. Insect Sci.* **2014**, *14*, 195.
48. Zeng, Z.; Wang, Y.; Johnson, N.A.N.; Wang, G.D.; Yao, Q.; Chen, K.P. Identification and phylogenetic analysis of basic helix-loop-helix genes in the diamondback moth. *J. Insect Sci.* **2018**, *18*,
49. Bitra, K.; Palli, S.R. The members of bHLH transcription factor superfamily are required for female reproduction in the red flour beetle, *Tribolium castaneum*. *J. Insect Physiol.* **2010**, *56*, 1481–1489.
50. Durand, B.; Drevet, J.; Couble, P. P25 gene regulation in *Bombyx mori* silk gland: two promoter-binding factors have distinct tissue and developmental specificities. *Mol. Cell. Biol.* **1992**, *12*, 5768–5777.
51. Peyrefitte, S.; Kahn, D.; Haenlin, M. New members of the *Drosophila* Myc transcription factor subfamily revealed by a genome-wide examination for basic helix-loop-helix genes. *Mech. Dev.* **2001**, *104*, 99–104.
52. Sassu, E.D.; McDermott, J.E.; Keys, B.J.; Esmaeili, M.; Keene, A.C.; Birnbaum, M.J.; DiAngelo, J.R. Mio/dChREBP coordinately increases fat mass by regulating lipid synthesis and feeding behavior in *Drosophila*. *Biochem. Biophys. Res. Commun.* **2012**, *426*, 43–48.
53. Chen, L.; Zhu, J.; Sun, G.; Raikhel, A.S. The early gene *Broad* is involved in the ecdysteroid hierarchy governing vitellogenesis of the mosquito *Aedes aegypti*. *J. Mol. Endocrinol.* **2004**, *33*, 743–761.
54. Zhu, J.; Chen, L.; Raikhel, A.S. Distinct roles of Broad isoforms in regulation of the 20-hydroxyecdysone effector gene, *Vitellogenin*, in the mosquito *Aedes aegypti*. *Mol. Cell Endocrinol.* **2007**, *267*, 97–105.
55. Cruz, J.; Mane-Padros, D.; Zou, Z.; Raikhel, A.S. Distinct roles of isoforms of the heme-liganded nuclear receptor E75, an insect ortholog of the vertebrate Rev-Erb, in mosquito reproduction. *Mol. Cell Endocrinol.* **2012**, *349*, 262–271.
56. Mane-Padros, D.; Cruz, J.; Cheng, A.; Raikhel, A.S. A critical role of the nuclear receptor HR3 in regulation of gonadotrophic cycles of the mosquito *Aedes aegypti*. *PLoS One* **2012**, *7*, e45019.
57. Cheng, D.; Qian, W.; Wang, Y.; Meng, M.; Wei, L.; Li, Z.; Kang, L.; Peng, J.; Xia, Q. Nuclear import of transcription factor BR-C is mediated by its interaction with RACK1. *PLoS One* **2014**, *9*, e109111.
58. Qian, W.; Gang, X.; Zhang, T.; Wei, L.; Yang, X.; Li, Z.; Yang, Y.; Song, L.; Wang, P.; Peng, J., et al. Protein kinase A-mediated phosphorylation of the Broad-Complex transcription factor in

silkworm suppresses its transcriptional activity. *J. Biol. Chem.* **2017**, *292*, 12460-12470.
59. Jiang, J.; Xu, Y.; Lin, X. Role of Broad-Complex (Br) and Krüppel homolog 1 (Kr-h1) in the Ovary Development of *Nilaparvata lugens*. *Front. Physiol.* **2017**, *8*, 1013.
60. Konopova, B.; Jindra, M. Broad-Complex acts downstream of Met in juvenile hormone signaling to coordinate primitive holometabolan metamorphosis. *Development* **2008**, *135*, 559–568.
61. Zhou, B.; Riddiford, L.M. Hormonal regulation and patterning of the broad-complex in the epidermis and wing discs of the tobacco hornworm, *Manduca sexta*. *Dev. Biol.* **2001**, *231*, 125–137.
62. Moeller, M.E.; Danielsen, E.T.; Herder, R.; O'Connor, M.B.; Rewitz, K.F. Dynamic feedback circuits function as a switch for shaping a maturation-inducing steroid pulse in *Drosophila*. *Development* **2013**, *140*, 4730–4739.
63. Yang, C.; Lin, Y.; Liu, H.; Shen, G.; Luo, J.; Zhang, H.; Peng, Z.; Chen, E.; Xing, R.; Han, C., et al. The Broad Complex isoform 2 (BrC-Z2) transcriptional factor plays a critical role in vitellogenin transcription in the silkworm *Bombyx mori*. *Biochim. Biophys. Acta* **2014**, *1840*, 2674–2684.
64. Lin, Y.; Liu, H.; Yang, C.; Gu, J.; Shen, G.; Zhang, H.; Chen, E.; Han, C.; Zhang, Y.; Xu, Y., et al. The POU homeodomain transcription factor POUM2 and broad complex isoform 2 transcription factor induced by 20-hydroxyecdysone collaboratively regulate vitellogenin gene expression and egg formation in the silkworm *Bombyx mori*. *Insect Mol. Biol.* **2017**, *26*, 496–506.
65. Wang, H.B.; Iwanaga, M.; Kawasaki, H. Activation of *Bm*WCP10 promoter and regulation by BR-C Z2 in wing disc of *Bombyx mori*. *Insect Biochem. Mol. Biol.* **2009**, *39*, 615–623.
66. Ali, M.S.; Rahman, R.F.; Swapon, A.H. Transcriptional regulation of cuticular protein glycine-rich13 gene expression in wing disc of *Bombyx mori*, Lepidoptera. *J. Insect Sci.* **2015**, *15*, 71–80.
67. Ali, M.S.; Mishra, B.; Rahman, R.F.; Swapon, A.H. The silkworm *Bombyx mori* cuticular protein *CPR55* gene is regulated by the transcription factor βFTZ-F1. *J. Insect Sci.* **2016**, *73*, 20–27.
68. Terashima, J.; Bownes, M. E75A and E75B have opposite effects on the apoptosis/development choice of the *Drosophila* egg chamber. *Cell Death Differ.* **2006**, *13*, 454–464.
69. Xiang, Y.; Liu, Z.; Huang, X. *br* regulates the expression of the ecdysone biosynthesis gene *npc1*. *Dev. Biol.* **2010**, *344*, 800–808.
70. Wang, H.B.; Nita, M.; Iwanaga, M.; Kawasaki, H. βFTZ-F1 and Broad-Complex positively regulate the transcription of the wing cuticle protein gene, *BmWCP5*, in wing discs of *Bombyx mori*. *Insect Biochem. Mol. Biol.* **2009**, *39*, 624–633.
71. Mai, T.; Chen, S.; Lin, X.; Zhang, X.; Zou, X.; Feng, Q.; Zheng, S. 20-hydroxyecdysone positively regulates the transcription of the antimicrobial peptide, lebocin, via BmEts and BmBR-C Z4 in the midgut of *Bombyx mori* during metamorphosis. *Dev. Comp. Immunol.* **2017**, *74*, 10–18.
72. Komori, H.; Xiao, Q.; Janssens, D.H.; Dou, Y.; Lee, C.Y. Trithorax maintains the functional heterogeneity of neural stem cells through the transcription factor buttonhead. *eLife* **2014**, *3*, e03502.
73. Chang, C.C.; Hsiao, Y.M.; Huang, T.Y.; Cook, C.E.; Shigenobu, S.; Chang, T.H. Noncanonical expression of *caudal* during early embryogenesis in the pea aphid *Acyrthosiphon pisum*: maternal cad-driven posterior development is not conserved. *Insect Mol. Biol.* **2013**, *22*, 442–455.
74. Macdonald, P.M.; Struhl, G. A molecular gradient in early *Drosophila* embryos and its role in specifying the body pattern. *Nature* **1986**, *324*, 537–545.
75. Dearolf, C.R.; Topol, J.; Parker, C.S. The *caudal* gene product is a direct activator of *fushi tarazu* transcription during *Drosophila* embryogenesis. *Nature* **1989**, *341*, 340–343.
76. Kühnlein, R.P.; Brönner, G.; Taubert, H.; Schuh, R. Regulation of *Drosophila spalt* gene expression. *Mech. Dev.* **1997**, *66*, 107–118.
77. Ryu, J.H.; Nam, K.B.; Oh, C.T.; Nam, H.J.; Kim, S.H.; Yoon, J.H.; Seong, J.K.; Yoo, M.A.; Jang, I.H.; Brey, P.T., et al. The homeobox gene *Caudal* regulates constitutive local expression of antimicrobial peptide genes in *Drosophila* epithelia. *Mol. Cell. Biol.* **2004**, *24*, 172–185.
78. Ryu, J.H.; Kim, S.H.; Lee, H.Y.; Bai, J.Y.; Nam, Y.D.; Bae, J.W.; Lee, D.G.; Shin, S.C.; Ha, E.M.; Lee, W.J. Innate immune homeostasis by the homeobox gene caudal and commensal-gut mutualism in *Drosophila*. *Science* **2008**, *319*, 777–782.
79. Junell, A.; Uvell, H.; Davis, M.M.; Edlundh-Rose, E.; Antonsson, A.; Pick, L.; Engström, Y. The

POU transcription factor Drifter/Ventral veinless regulates expression of *Drosophila* immune defense genes. *Mol. Cell Biol.* **2010**, *30*, 3672–3684.
80. Nakao, H. Anterior and posterior centers jointly regulate *Bombyx* embryo body segmentation. *Dev. Biol.* **2012**, *371*, 293–301.
81. Goulding, S.E.; White, N.M.; Jarman, A.P. *cato* encodes a basic helix-loop-helix transcription factor implicated in the correct differentiation of *Drosophila* sense organs. *Dev. Biol.* **2000**, *221*, 120–131.
82. Sourmeli, S.; Papantonis, A.; Lecanidou, R. BmCbZ, an insect-specific factor featuring a composite DNA-binding domain, interacts with BmC/EBPγ. *Biochem. Biophys. Res. Commun.* **2005**, *338*, 1957–1965.
83. Montell, D.J.; Rorth, P.; Spradling, A.C. *slow border cells*, a locus required for a developmentally regulated cell migration during oogenesis, encodes *Drosophila* C/EBP. *Cell* **1992**, *71*, 51–62.
84. Levine, B.; Hackney, J.F.; Bergen, A.; Dobens, L., 3rd; Truesdale, A.; Dobens, L. Opposing interactions between *Drosophila* Cut and the C/EBP encoded by slow border cells direct apical constriction and epithelial invagination. *Dev. Biol.* **2010**, *344*, 196–209.
85. Masoner, V.; Das, R.; Pence, L.; Anand, G.; LaFerriere, H.; Zars, T.; Bouyain, S.; Dobens, L.L. The kinase domain of *Drosophila* Tribbles is required for turnover of fly C/EBP during cell migration. *Dev. Biol.* **2013**, *375*, 33–44.
86. Sourmeli, S.; Papantonis, A.; Lecanidou, R. A novel role for the *Bombyx* Slbo homologue, BmC/EBP, in insect choriogenesis. *Biochem. Biophys. Res. Commun.* **2005**, *337*, 713–719.
87. Papantonis, A.; Sourmeli, S.; Lecanidou, R. Chorion gene activation and repression is dependent on BmC/EBP expression and binding to cognate *cis*-elements. *Biochem. Biophys. Res. Commun.* **2008**, *369*, 905–909.
88. Meredith, J.M.; Munks, R.J.; Grail, W.; Hurd, H.; Eggleston, P.; Lehane, M.J. A novel association between clustered NF-κB and C/EBP binding sites is required for immune regulation of mosquito *Defensin* genes. *Insect Mol. Biol.* **2006**, *15*, 393–401.
89. Liang, L.N.; Zhang, L.L.; Zeng, B.J.; Zheng, S.C.; Feng, Q.L. Transcription factor CAAT/enhancer-binding protein is involved in regulation of expression of sterol carrier protein x in *Spodoptera litura*. *Insect Mol. Biol.* **2015**, *24*, 551–560.
90. McGinnis, N.; Ragnhildstveit, E.; Veraksa, A.; McGinnis, W. A cap 'n' collar protein isoform contains a selective Hox repressor function. *Development* **1998**, *125*, 4553–4564.
91. Deng, H.; Kerppola, T.K. Regulation of *Drosophila* metamorphosis by xenobiotic response regulators. *PLoS Genet.* **2013**, *9*, e1003263.
92. Sykiotis, G.P.; Bohmann, D. Keap1/Nrf2 signaling regulates oxidative stress tolerance and lifespan in *Drosophila*. *Dev. Cell* **2008**, *14*, 76–85.
93. Jain, A.; Rusten, T.E.; Katheder, N.; Elvenes, J.; Bruun, J.A.; Sjøttem, E.; Lamark, T.; Johansen, T. p62/sequestosome-1, autophagy-related Gene 8, and autophagy in *Drosophila* are regulated by nuclear factor erythroid 2-related factor 2 (NRF2), independent of transcription factor TFEB. *J. Biol. Chem.* **2015**, *290*, 14945–14962.
94. Misra, J.R.; Horner, M.A.; Lam, G.; Thummel, C.S. Transcriptional regulation of xenobiotic detoxification in *Drosophila*. *Genes Dev.* **2011**, *25*, 1796–1806.
95. Misra, J.R.; Lam, G.; Thummel, C.S. Constitutive activation of the Nrf2/Keap1 pathway in insecticide-resistant strains of *Drosophila*. *Insect Biochem. Mol. Biol.* **2013**, *43*, 1116–1124.
96. Wan, H.; Liu, Y.; Li, M.; Zhu, S.; Li, X.; Pittendrigh, B.R.; Qiu, X. Nrf2/Maf-binding-site-containing functional *Cyp6a2* allele is associated with DDT resistance in *Drosophila melanogaster*. *Pest Manag. Sci.* **2014**, *70*, 1048–1058.
97. Guio, L.; Barron, M.G.; González, J. The transposable element *Bari-Jheh* mediates oxidative stress response in *Drosophila*. *Mol. Ecol.* **2014**, *23*, 2020–2030.
98. Pitoniak, A.; Bohmann, D. Mechanisms and functions of Nrf2 signaling in *Drosophila*. *Free Radic. Biol. Med.* **2015**, *88*, 302–313.
99. Wilding, C.S. Regulating resistance: CncC:Maf, antioxidant response elements and the overexpression of detoxification genes in insecticide resistance. *Curr. Opin. Insect Sci.* **2018**, *27*, 89–96.
100. Kalsi, M.; Palli, S.R. Transcription factors, CncC and Maf, regulate expression of CYP6BQ genes

responsible for deltamethrin resistance in *Tribolium castaneum*. *Insect Biochem. Mol. Biol.* **2015**, *65*, 47–56.

101. Kalsi, M.; Palli, S.R. Cap n collar transcription factor regulates multiple genes coding for proteins involved in insecticide detoxification in the red flour beetle, *Tribolium castaneum*. *Insect Biochem. Mol. Biol.* **2017**, *90*, 43–52.
102. Kalsi, M.; Palli, S.R. Transcription factor cap n collar C regulates multiple cytochrome P450 genes conferring adaptation to potato plant allelochemicals and resistance to imidacloprid in *Leptinotarsa decemlineata* (Say). *Insect Biochem. Mol. Biol.* **2017**, *83*, 1–12.
103. Peng, T.; Pan, Y.; Gao, X.; Xi, J.; Zhang, L.; Yang, C.; Bi, R.; Yang, S.; Xin, X.; Shang, Q. Cytochrome P450 *CYP6DA2* regulated by *cap 'n'collar isoform C* (*CncC*) is associated with gossypol tolerance in *Aphis gossypii* Glover. *Insect Mol. Biol.* **2016**, *25*, 450–459.
104. Hu, J.; Chen, J.; Wang, H.; Mao, T.; Li, J.; Cheng, X.; Hu, J.; Xue, B.; Li, B. Cloning and functional analysis of CncC and Keap1 genes in silkworm. *J. Agric. Food Chem.* **2018**, *66*, 2630–2636.
105. Cheng, X.; Hu, J.; Li, J.; Chen, J.; Wang, H.; Mao, T.; Xue, B.; Li, B. The silk gland damage and the transcriptional response to detoxifying enzymes-related genes of *Bombyx mori* under phoxim exposure. *Chemosphere* **2018**, *209*, 964–971.
106. Crozatier, M.; Valle, D.; Dubois, L.; Ibnsouda, S.; Vincent, A. *collier*, a novel regulator of *Drosophila* head development, is expressed in a single mitotic domain. *Curr. Biol.* **1996**, *6*, 707–718.
107. Dubois, L.; Enriquez, J.; Daburon, V.; Crozet, F.; Lebreton, G.; Crozatier, M.; Vincent, A. Collier transcription in a single *Drosophila* muscle lineage: the combinatorial control of muscle identity. *Development* **2007**, *134*, 4347–4355.
108. Crozatier, M.; Vincent, A. Control of multidendritic neuron differentiation in *Drosophila*: the role of Collier. *Dev. Biol.* **2008**, *315*, 232–242.
109. Enriquez, J.; de Taffin, M.; Crozatier, M.; Vincent, A.; Dubois, L. Combinatorial coding of *Drosophila* muscle shape by Collier and Nautilus. *Dev. Biol.* **2012**, *363*, 27–39.
110. Benmimoun, B.; Polesello, C.; Haenlin, M.; Waltzer, L. The EBF transcription factor Collier directly promotes *Drosophila* blood cell progenitor maintenance independently of the niche. *Proc. Natl. Acad. Sci. U. S. A.* **2015**, *112*, 9052–9057.
111. Hewes, R.S.; Schaefer, A.M.; Taghert, P.H. The *cryptocephal* gene (ATF4) encodes multiple basic-leucine zipper proteins controlling molting and metamorphosis in *Drosophila*. *Genetics* **2000**, *155*, 1711–1723.
112. Gauthier, S.A.; Hewes, R.S. Transcriptional regulation of neuropeptide and peptide hormone expression by the *Drosophila dimmed* and *cryptocephal* genes. *J. Exp. Biol.* **2006**, *209*, 1803–1815.
113. Gauthier, S.A.; VanHaaften, E.; Cherbas, L.; Cherbas, P.; Hewes, R.S. Cryptocephal, the *Drosophila melanogaster* ATF4, is a specific coactivator for ecdysone receptor isoform B2. *PLoS Genet.* **2012**, *8*, e1002883.
114. Belvin, M.P.; Zhou, H.; Yin, J.C. The *Drosophila dCREB2* gene affects the circadian clock. *Neuron* **1999**, *22*, 777–787.
115. Tanenhaus, A.K.; Zhang, J.; Yin, J.C. *In vivo* circadian oscillation of dCREB2 and NF-κB activity in the *Drosophila* nervous system. *PLoS One* **2012**, *7*, e45130.
116. Zhang, J.; Tanenhaus, A.K.; Davis, J.C.; Hanlon, B.M.; Yin, J.C. Spatio-temporal *in vivo* recording of dCREB2 dynamics in *Drosophila* long-term memory processing. *Neurobiol. Learn. Mem.* **2015**, *118*, 80–88.
117. Song, H.; Sun, Y.; Zhang, Y.; Li, M. Molecular cloning and characterization of *Bombyx mori* CREB gene. *Arch. Insect Biochem. Physiol.* **2009**, *71*, 31–44.
118. Liu, L.; Qian, X.; Chao, M.; Zhao, Y.; Huang, J.; Wang, T.; Sun, F.; Ling, E.; Song, H. Aluminum toxicity related to SOD and expression of presenilin and CREB in *Bombyx mori*. *Arch. Insect Biochem. Physiol.* **2018**, *99*, e21480.
119. van den Berg, M.; Verbaarschot, P.; Hontelez, S.; Vet, L.E.; Dicke, M.; Smid, H.M. CREB expression in the brains of two closely related parasitic wasp species that differ in long-term memory formation. *Insect Mol. Biol.* **2010**, *19*, 367–379.
120. Gehring, K.B.; Heufelder, K.; Kersting, I.; Eisenhardt, D. Abundance of phosphorylated *Apis mellifera* CREB in the honeybee's mushroom body inner compact cells varies with age. *J. Comp.*

*Neurol.* **2016**, *524*, 1165–1180.
121. Gehring, K.B.; Heufelder, K.; Feige, J.; Bauer, P.; Dyck, Y.; Ehrhardt, L.; Kühnemund, J.; Bergmann, A.; Göbel, J.; Isecke, M., et al. Involvement of phosphorylated *Apis mellifera* CREB in gating a honeybee's behavioral response to an external stimulus. *Learn. Mem.* **2016**, *23*, 195–207.
122. Gao, J.; Sun, Y.; Sun, Y.; Chen, C.; Kausar, S.; Tian, J.; Zhu, B.; Liu, C. Identification and function of cAMP response element binding protein in Oak silkworm *Antheraea pernyi*. *J. Invertebr. Pathol.* **2018**, *151*, 14–20.
123. Abrams, E.W.; Andrew, D.J. CrebA regulates secretory activity in the *Drosophila* salivary gland and epidermis. *Development* **2005**, *132*, 2743–2758.
124. Fox, R.M.; Hanlon, C.D.; Andrew, D.J. The CrebA/Creb3-like transcription factors are major and direct regulators of secretory capacity. *J. Cell Biol.* **2010**, *191*, 479–492.
125. Iyer, S.C.; Ramachandran Iyer, E.P.; Meduri, R.; Rubaharan, M.; Kuntimaddi, A.; Karamsetty, M.; Cox, D.N. Cut, via CrebA, transcriptionally regulates the COPII secretory pathway to direct dendrite development in *Drosophila*. *J. Cell Sci.* **2013**, *126*, 4732–4745.
126. Kang, K.; Yang, P.; Pang, R.; Yue, L.; Zhang, W. *Cycle* affects imidacloprid efficiency by mediating cytochrome P450 expression in the brown planthopper *Nilaparvata lugens*. *Insect Mol. Biol.* **2017**, *26*, 522–529.
127. Rutila, J.E.; Suri, V.; Le, M.; So, W.V.; Rosbash, M.; Hall, J.C. CYCLE is a second bHLH-PAS clock protein essential for circadian rhythmicity and transcription of *Drosophila period* and *timeless*. *Cell* **1998**, *93*, 805–814.
128. Bae, K.; Lee, C.; Hardin, P.E.; Edery, I. dCLOCK is present in limiting amounts and likely mediates daily interactions between the dCLOCK-CYC transcription factor and the PER-TIM complex. *J. Neurosci.* **2000**, *20*, 1746–1753.
129. Glossop, N.R.; Houl, J.H.; Zheng, H.; Ng, F.S.; Dudek, S.M.; Hardin, P.E. VRILLE feeds back to control circadian transcription of *Clock* in the *Drosophila* circadian oscillator. *Neuron* **2003**, *37*, 249–261.
130. Cyran, S.A.; Buchsbaum, A.M.; Reddy, K.L.; Lin, M.C.; Glossop, N.R.; Hardin, P.E.; Young, M.W.; Storti, R.V.; Blau, J. *vrille*, *Pdp1*, and *dClock* form a second feedback loop in the *Drosophila* circadian clock. *Cell* **2003**, *112*, 329–341.
131. Bajgar, A.; Jindra, M.; Dolezel, D. Autonomous regulation of the insect gut by circadian genes acting downstream of juvenile hormone signaling. *Proc. Natl. Acad. Sci. U. S. A.* **2013**, *110*, 4416–4421.
132. Matsumoto, A.; Ukai-Tadenuma, M.; Yamada, R.G.; Houl, J.; Uno, K.D.; Kasukawa, T.; Dauwalder, B.; Itoh, T.Q.; Takahashi, K.; Ueda, R., et al. A functional genomics strategy reveals *clockwork orange* as a transcriptional regulator in the *Drosophila* circadian clock. *Genes Dev.* **2007**, *21*, 1687–1700.
133. Brown, N.L.; Paddock, S.W.; Sattler, C.A.; Cronmiller, C.; Thomas, B.J.; Carroll, S.B. *daughterless* is required for *Drosophila* photoreceptor cell determination, eye morphogenesis, and cell cycle progression. *Dev. Biol.* **1996**, *179*, 65–78.
134. Andrade-Zapata, I.; Baonza, A. The bHLH factors Extramacrochaetae and Daughterless control cell cycle in *Drosophila* imaginal discs through the transcriptional regulation of the *cdc25* phosphatase *string*. *PLoS Genet.* **2014**, *10*, e1004233.
135. Miguel-Aliaga, I.; Allan, D.W.; Thor, S. Independent roles of the *dachshund* and *eyes absent* genes in BMP signaling, axon pathfinding and neuronal specification. *Development* **2004**, *131*, 5837–5848.
136. Pérez-Zamorano, B.; Rosas-Madrigal, S.; Lozano, O.A.M.; Castillo Méndez, M.; Valverde-Garduño, V. Identification of *cis*-regulatory sequences reveals potential participation of lola and Deaf1 transcription factors in *Anopheles gambiae* innate immune response. *PLoS One* **2017**, *12*, e0186435.
137. Armand, P.; Knapp, A.C.; Hirsch, A.J.; Wieschaus, E.F.; Cole, M.D. A novel basic helix-loop-helix protein is expressed in muscle attachment sites of the *Drosophila* epidermis. *Mol. Cell. Biol.* **1994**, *14*, 4145–4154.
138. Egoz-Matia, N.; Nachman, A.; Halachmi, N.; Toder, M.; Klein, Y.; Salzberg, A. Spatial regulation of cell adhesion in the *Drosophila* wing is mediated by Delilah, a potent activator of βPS integrin expression. *Dev. Biol.* **2011**, *351*, 99–109.
139. Shen, S.P.; Aleksic, J.; Russell, S. Identifying targets of the Sox domain protein Dichaete in the

*Drosophila* CNS via targeted expression of dominant negative proteins. *BMC Dev. Biol.* **2013**, *13*, 1.
140. Zambon, R.A.; Nandakumar, M.; Vakharia, V.N.; Wu, L.P. The Toll pathway is important for an antiviral response in *Drosophila*. *Proc. Natl. Acad. Sci. U. S. A.* **2005**, *102*, 7257–7262.
141. Tanji, T.; Yun, E.Y.; Ip, Y.T. Heterodimers of NF-κB transcription factors DIF and Relish regulate antimicrobial peptide genes in *Drosophila*. *Proc. Natl. Acad. Sci. U. S. A.* **2010**, *107*, 14715–14720.
142. Park, D.; Han, M.; Kim, Y.C.; Han, K.A.; Taghert, P.H. Ap-let neurons--a peptidergic circuit potentially controlling ecdysial behavior in *Drosophila*. *Dev. Biol.* **2004**, *269*, 95–108.
143. Park, D.; Shafer, O.T.; Shepherd, S.P.; Suh, H.; Trigg, J.S.; Taghert, P.H. The *Drosophila* basic helix-loop-helix protein DIMMED directly activates *PHM*, a gene encoding a neuropeptide-amidating enzyme. *Mol. Cell Biol.* **2008**, *28*, 410–421.
144. Park, D.; Veenstra, J.A.; Park, J.H.; Taghert, P.H. Mapping peptidergic cells in *Drosophila*: where DIMM fits in. *PLoS One* **2008**, *3*, e1896.
145. Park, D.; Hadžić, T.; Yin, P.; Rusch, J.; Abruzzi, K.; Rosbash, M.; Skeath, J.B.; Panda, S.; Sweedler, J.V.; Taghert, P.H. Molecular organization of *Drosophila* neuroendocrine cells by *Dimmed*. *Curr. Biol.* **2011**, *21*, 1515–1524.
146. Hadžić, T.; Park, D.; Abruzzi, K.C.; Yang, L.; Trigg, J.S.; Rohs, R.; Rosbash, M.; Taghert, P.H. Genome-wide features of neuroendocrine regulation in *Drosophila* by the basic helix-loop-helix transcription factor DIMMED. *Nucleic Acids Res.* **2015**, *43*, 2199–2215.
147. Beebe, K.; Park, D.; Taghert, P.H.; Micchelli, C.A. The *Drosophila* prosecretory transcription factor *dimmed* is dynamically regulated in adult enteroendocrine cells and protects against Gram-negative infection. In *G3*, 2015; Vol. 5, pp 1517–1524.
148. Zhao, X.M.; Liu, C.; Jiang, L.J.; Li, Q.Y.; Zhou, M.T.; Cheng, T.C.; Mita, K.; Xia, Q.Y. A juvenile hormone transcription factor Bmdimm-fibroin H chain pathway is involved in the synthesis of silk protein in silkworm, *Bombyx mori*. *J. Biol. Chem.* **2015**, *290*, 972–986.
149. Zhou, C.; Zha, X.; Shi, P.; Wei, S.; Wang, H.; Zheng, R.; Xia, Q. Multiprotein bridging factor 2 regulates the expression of the *fibroin heavy chain* gene by interacting with Bmdimmed in the silkworm *Bombyx mori*. *Insect Mol. Biol.* **2016**, *25*, 509–518.
150. Diederich, R.J.; Pattatucci, A.M.; Kaufman, T.C. Developmental and evolutionary implications of labial, Deformed and engrailed expression in the *Drosophila* head. *Development* **1991**, *113*, 273–281.
151. Ratnaparkhi, G.S.; Duong, H.A.; Courey, A.J. Dorsal interacting protein 3 potentiates activation by *Drosophila* Rel homology domain proteins. *Dev. Comp. Immunol.* **2008**, *32*, 1290–1300.
152. Zehavi, Y.; Kuznetsov, O.; Ovadia-Shochat, A.; Juven-Gershon, T. Core promoter functions in the regulation of gene expression of *Drosophila* dorsal target genes. *J. Biol. Chem.* **2014**, *289*, 11993–12004.
153. Zhong, X.; Rao, X.J.; Yi, H.Y.; Lin, X.Y.; Huang, X.H.; Yu, X.Q. Co-expression of Dorsal and Rel2 negatively regulates antimicrobial peptide expression in the tobacco hornworm *Manduca sexta*. *Sci. Rep.* **2016**, *6*, 20654.
154. Kappes, G.; Deshpande, G.; Mulvey, B.B.; Horabin, J.I.; Schedl, P. The *Drosophila* Myc gene, *diminutive*, is a positive regulator of the *Sex-lethal* establishment promoter, *Sxl-Pe*. *Proc. Natl. Acad. Sci. U. S. A.* **2011**, *108*, 1543–1548.
155. Segev, E.; Halachmi, N.; Salzberg, A.; Ben-Arie, N. Nato3 is an evolutionarily conserved bHLH transcription factor expressed in the CNS of *Drosophila* and mouse. *Mech. Dev.* **2001**, *106*, 197–202.
156. Younger-Shepherd, S.; Vaessin, H.; Bier, E.; Jan, L.Y.; Jan, Y.N. *deadpan*, an essential pan-neural gene encoding an HLH protein, acts as a denominator in *Drosophila* sex determination. *Cell* **1992**, *70*, 911–922.
157. Lu, H.; Kozhina, E.; Mahadevaraju, S.; Yang, D.; Avila, F.W.; Erickson, J.W. Maternal Groucho and bHLH repressors amplify the dose-sensitive X chromosome signal in *Drosophila* sex determination. *Dev. Biol.* **2008**, *323*, 248–260.
158. San-Juán, B.P.; Baonza, A. The bHLH factor *deadpan* is a direct target of Notch signaling and regulates neuroblast self-renewal in *Drosophila*. *Dev. Biol.* **2011**, *352*, 70–82.
159. Zhu, S.; Wildonger, J.; Barshow, S.; Younger, S.; Huang, Y.; Lee, T. The bHLH repressor Deadpan regulates the self-renewal and specification of *Drosophila* larval neural stem cells independently of Notch. *PLoS One* **2012**, *7*, e46724.

160. Janssens, D.H.; Hamm, D.C.; Anhezini, L.; Xiao, Q.; Siller, K.H.; Siegrist, S.E.; Harrison, M.M.; Lee, C.Y. An Hdac1/Rpd3-poised circuit balances continual self-renewal and rapid restriction of developmental potential during asymmetric stem cell division. *Dev. Cell* **2017**, *40*, 367–380.
161. Wallace, K.; Liu, T.H.; Vaessin, H. The pan-neural bHLH proteins DEADPAN and ASENSE regulate mitotic activity and cdk inhibitor *dacapo* expression in the *Drosophila* larval optic lobes. *Genesis* **2000**, *26*, 77–85.
162. Mavromatakis, Y.E.; Tomlinson, A. R7 photoreceptor specification in the developing *Drosophila* eye: The role of the transcription factor Deadpan. *PLoS Genet.* **2016**, *12*, e1006159.
163. Mysore, K.; Sun, L.; Tomchaney, M.; Sullivan, G.; Adams, H.; Piscoya, A.S.; Severson, D.W.; Syed, Z.; Duman-Scheel, M. siRNA-mediated silencing of *doublesex* during female development of the dengue vector mosquito *Aedes aegypti*. *PLoS Negl. Trop. Dis.* **2015**, *9*, e0004213.
164. Shukla, J.N.; Nagaraju, J. Two female-specific DSX proteins are encoded by the sex-specific transcripts of *dsx*, and are required for female sexual differentiation in two wild silkmoth species, *Antheraea assama* and *Antheraea mylitta* (Lepidoptera, Saturniidae). *Insect Biochem. Mol. Biol.* **2010**, *40*, 672–682.
165. Velasque, M.; Qiu, L.; Mikheyev, A.S. The *Doublesex* sex determination pathway regulates reproductive division of labor in honey bees. *bioRxiv* **2018**, 10.1101/314492.
166. Duan, J.; Xu, H.; Wang, F.; Ma, S.; Zha, X.; Guo, H.; Zhao, P.; Xia, Q. Novel female-specific trans-spliced and alternative splice forms of *dsx* in the silkworm *Bombyx mori*. *Biochem. Biophys. Res. Commun.* **2013**, *431*, 6306–6305.
167. Duan, J.; Xu, H.; Guo, H.; O'Brochta, D.A.; Wang, F.; Ma, S.; Zhang, L.; Zha, X.; Zhao, P.; Xia, Q. New insights into the genomic organization and splicing of the *doublesex* gene, a terminal regulator of sexual differentiation in the silkworm *Bombyx mori*. *PLoS One* **2013**, *8*, e79703.
168. Xu, J.; Zhan, S.; Chen, S.; Zeng, B.; Li, Z.; James, A.A.; Tan, A.; Huang, Y. Sexually dimorphic traits in the silkworm, *Bombyx mori*, are regulated by *doublesex*. *Insect Biochem. Mol. Biol.* **2017**, *80*, 42–51.
169. Gotoh, H.; Miyakawa, H.; Ishikawa, A.; Ishikawa, Y.; Sugime, Y.; Emlen, D.J.; Lavine, L.C.; Miura, T. Developmental link between sex and nutrition; *doublesex* regulates sex-specific mandible growth via juvenile hormone signaling in stag beetles. *PLoS Genet.* **2014**, *10*, e1004098.
170. Burtis, K.C.; Coschigano, K.T.; Baker, B.S.; Wensink, P.C. The doublesex proteins of *Drosophila melanogaster* bind directly to a sex-specific yolk protein gene enhancer. *EMBO J.* **1991**, *10*, 2577–2582.
171. Shirangi, T.R.; Dufour, H.D.; Williams, T.M.; Carroll, S.B. Rapid evolution of sex pheromone-producing enzyme expression in *Drosophila*. *PLoS Biol.* **2009**, *7*, e1000168.
172. Williams, T.M.; Selegue, J.E.; Werner, T.; Gompel, N.; Kopp, A.; Carroll, S.B. The regulation and evolution of a genetic switch controlling sexually dimorphic traits in *Drosophila*. *Cell* **2008**, *134*, 610–623.
173. Luo, S.D.; Shi, G.W.; Baker, B.S. Direct targets of the *D. melanogaster* DSXF protein and the evolution of sexual development. *Development* **2011**, *138*, 2761–2771.
174. Ledón-Rettig, C.C.; Zattara, E.E.; Moczek, A.P. Asymmetric interactions between doublesex and tissue- and sex-specific target genes mediate sexual dimorphism in beetles. *Nat. Commun.* **2017**, *8*, 14593.
175. Rice, G.; Barmina, O.; Hu, K.; Kopp, A. Evolving *doublesex* expression correlates with the origin and diversification of male sexual ornaments in the *Drosophila immigrans* species group. *Evol. Dev.* **2018**, *20*, 78–88.
176. Zhuo, J.C.; Hu, Q.L.; Zhang, H.H.; Zhang, M.Q.; Jo, S.B.; Zhang, C.X. Identification and functional analysis of the *doublesex* gene in the sexual development of a hemimetabolous insect, the brown planthopper. *Insect Biochem. Mol. Biol.* **2018**, *102*, 31–42.
177. Beckers, O.M.; Kijimoto, T.; Moczek, A.P. *doublesex* alters aggressiveness as a function of social context and sex in the polyphenic beetle *Onthophagus taurus*. *Anim. Behav.* **2017**, *132*, 261–269.
178. Sugimoto, T.N.; Fujii, T.; Kayukawa, T.; Sakamoto, H.; Ishikawa, Y. Expression of a *doublesex* homologue is altered in sexual mosaics of *Ostrinia scapulalis* moths infected with Wolbachia. *Insect Biochem. Mol. Biol.* **2010**, *40*, 847–854.
179. Ruiz, M.F.; Alvarez, M.; Eirin-Lopez, J.M.; Sarno, F.; Kremer, L.; Barbero, J.L.; Sanchez, L. An

unusual role for *doublesex* in sex determination in the Dipteran *Sciara*. *Genetics* **2015**, *200*, 1181–1199.

180. Du, Q.; Wen, L.; Zheng, S.C.; Bi, H.L.; Huang, Y.P.; Feng, Q.L.; Liu, L. Identification and functional characterization of *doublesex* gene in the testis of *Spodoptera litura*. *Insect Sci.* **2018**, 10.1111/1744-7917.12608.
181. Shukla, J.N.; Palli, S.R. Doublesex target genes in the red flour beetle, *Tribolium castaneum*. *Sci. Rep.* **2012**, *2*, 948.
182. Ito, Y.; Harigai, A.; Nakata, M.; Hosoya, T.; Araya, K.; Oba, Y.; Ito, A.; Ohde, T.; Yaginuma, T.; Niimi, T. The role of *doublesex* in the evolution of exaggerated horns in the Japanese rhinoceros beetle. *EMBO Rep.* **2013**, *14*, 561–567.
183. Morita, S.; Ando, T.; Maeno, A.; Mizutani, T.; Mase, M.; Shigenobu, S.; Niimi, T. *doublesex* regulates sexually dimorphic beetle horn formation by integrating spatial and temporal developmental contexts in the Japanese rhinoceros beetle *Trypoxylus dichotomus*. *bioRxiv* **2018**, 10.1101/328120.
184. Jiang, L.; Crews, S.T. The *Drosophila dysfusion* basic helix-loop-helix (bHLH)-PAS gene controls tracheal fusion and levels of the trachealess bHLH-PAS protein. *Mol. Cell Biol.* **2003**, *23*, 5625–5637.
185. Jiang, L.; Crews, S.T. *dysfusion* transcriptional control of *Drosophila* tracheal migration, adhesion, and fusion. *Mol. Cell Biol.* **2006**, *26*, 6547–6556.
186. Jiang, L.; Crews, S.T. Transcriptional specificity of *Drosophila* Dysfusion and the control of tracheal fusion cell gene expression. *J. Biol. Chem.* **2007**, *282*, 28659–28668.
187. Córdoba, S.; Estella, C. The bHLH-PAS transcription factor Dysfusion regulates tarsal joint formation in response to Notch activity during *Drosophila* leg development. *PLoS Genet.* **2014**, *10*, e1004621.
188. Abbas, M.N.; Kausar, S.; Sun, Y.X.; Sun, Y.; Wang, L.; Qian, C.; Wei, G.Q.; Zhu, B.J.; Liu, C.L. Molecular cloning, expression, and characterization of E2F transcription factor 4 from *Antheraea pernyi*. *Bull. Entomol. Res.* **2017**, *107*, 839–846.
189. Tian, L.; Ma, L.; Guo, E.; Deng, X.; Ma, S.; Xia, Q.; Cao, Y.; Li, S. 20-Hydroxyecdysone upregulates Atg genes to induce autophagy in the *Bombyx* fat body. *Autophagy* **2013**, *9*, 1172–1187.
190. Sun, W.; Wang, C.F.; Zhang, Z. Transcription factor E74A affects the ecdysone titer by regulating the expression of the EO gene in the silkworm, *Bomby mori*. *Biochim. Biophys. Acta* **2017**, *1861*, 551–558.
191. Frank, H.O.; Sanchez, D.G.; de Freitas Oliveira, L.; Kobarg, J.; Monesi, N. The *Drosophila melanogaster* Eip74EF-PA transcription factor directly binds the sciarid *BhC4-1* promoter. *Genesis* **2017**, *55*, e23075.
192. Sun, G.; Zhu, J.; Raikhel, A.S. The early gene E74B isoform is a transcriptional activator of the ecdysteroid regulatory hierarchy in mosquito vitellogenesis. *Mol. Cell Endocrinol.* **2004**, *218*, 95–105.
193. Li, K.; Tian, L.; Guo, Z.; Guo, S.; Zhang, J.; Gu, S.H.; Palli, S.R.; Cao, Y.; Li, S. 20-hydroxyecdysone (20E) primary response gene *E75* isoforms mediate steroidogenesis autoregulation and regulate developmental timing in *Bombyx*. *J. Biol. Chem.* **2016**, *291*, 18163–18175.
194. Kamae, Y.; Uryu, O.; Miki, T.; Tomioka, K. The nuclear receptor genes *HR3* and *E75* are required for the circadian rhythm in a primitive insect. *PLoS One* **2014**, *9*, e114899.
195. Jaumouillé, E.; Machado Almeida, P.; Stähli, P.; Koch, R.; Nagoshi, E. Transcriptional regulation via nuclear receptor crosstalk required for the *Drosophila* circadian clock. *Curr. Biol.* **2015**, *25*, 1502–1508.
196. Cao, C.; Liu, Y.; Lehmann, M. Fork head controls the timing and tissue selectivity of steroid-induced developmental cell death. *J. Cell Biol.* **2007**, *176*, 843–852.
197. Hiruma, K.; Riddiford, L.M. Differential control of MHR3 promoter activity by isoforms of the ecdysone receptor and inhibitory effects of E75A and MHR3. *Dev. Biol.* **2004**, *272*, 510–521.
198. Hiruma, K.; Riddiford, L.M. The coordination of the sequential appearance of MHR4 and dopa decarboxylase during the decline of the ecdysteroid titer at the end of the molt. *Mol. Cell Endocrinol.* **2007**, *276*, 71–79.
199. Siaussat, D.; Bozzolan, F.; Queguiner, I.; Porcheron, P.; Debernard, S. Effects of juvenile hormone on 20-hydroxyecdysone-inducible EcR, HR3, E75 gene expression in imaginal wing cells of *Plodia interpunctella* lepidoptera. *Eur. J. Biochem.* **2004**, *271*, 3017–3027.

200. Liu, X.; Dai, F.; Guo, E.; Li, K.; Ma, L.; Tian, L.; Cao, Y.; Zhang, G.; Palli, S.R.; Li, S. 20-hydroxyecdysone (20E) primary response gene E93 modulates 20E signaling to promote *Bombyx* larval-pupal metamorphosis. *J. Biol. Chem.* **2015**, *290*, 27370–27383.
201. Chafino, S.; López-Escardó, D.; Benelli, G.; Kovac, H.; Casacuberta, E.; Franch-Marro, X.; Kathirithamby, J.; Martín, D. Differential expression of the adult specifier E93 in the strepsipteran *Xenos vesparum* Rossi suggests a role in female neoteny. *Sci. Rep.* **2018**, *8*, 14176.
202. Goncu, E.; Parlak, O. Morphological changes and patterns of ecdysone receptor B1 immunolocalization in the anterior silk gland undergoing programmed cell death in the silkworm, *Bombyx mori*. *Acta Histochem.* **2009**, *111*, 25–34.
203. Wang, H.B.; Moriyama, M.; Iwanaga, M.; Kawasaki, H. Ecdysone directly and indirectly regulates a cuticle protein gene, *BMWCP10*, in the wing disc of *Bombyx mori*. *Insect Biochem. Mol. Biol.* **2010**, *40*, 453–459.
204. Monteiro, A.; Tong, X.; Bear, A.; Liew, S.F.; Bhardwaj, S.; Wasik, B.R.; Dinwiddie, A.; Bastianelli, C.; Cheong, W.F.; Wenk, M.R., et al. Differential expression of ecdysone receptor leads to variation in phenotypic plasticity across serial homologs. *PLoS Genet.* **2015**, *11*, e1005529.
205. Fichelson, P.; Brigui, A.; Pichaud, F. Orthodenticle and Kruppel homolog 1 regulate *Drosophila* photoreceptor maturation. *Proc. Natl. Acad. Sci. U. S. A.* **2012**, *109*, 7893–7898.
206. King-Jones, K.; Thummel, C.S. Nuclear receptors–a perspective from *Drosophila*. *Nat. Rev. Genet.* **2005**, *6*, 311–323.
207. Lenaerts, C.; Van Wielendaele, P.; Peeters, P.; Vanden Broeck, J.; Marchal, E. Ecdysteroid signalling components in metamorphosis and development of the desert locust, *Schistocerca gregaria*. *Insect Biochem. Mol. Biol.* **2016**, *75*, 10–23.
208. Van Doren, M.; Ellis, H.M.; Posakony, J.W. The *Drosophila extramacrochaetae* protein antagonizes sequence-specific DNA binding by *daughterless/achaete-scute* protein complexes. *Development* **1991**, *113*, 245–255.
209. Spratford, C.M.; Kumar, J.P. Extramacrochaetae functions in dorsal-ventral patterning of *Drosophila* imaginal discs. *Development* **2015**, *142*, 1006–1015.
210. Liu, W.; Chai, D.; Wang, C.; Li, Q.; Lei, J.; Yang, M.; Dai, F.; Lu, C. The *extramacrochaetae* gene is required for blastokinesis in silkworm, *Bombyx mori*. *J. Exp. Zool.* **2015**, *324B*, 405–409.
211. Sen, S.; Hartmann, B.; Reichert, H.; Rodrigues, V. Expression and function of the empty spiracles gene in olfactory sense organ development of *Drosophila melanogaster*. *Development* **2010**, *137*, 3687–3695.
212. Luo, L.; Siah, C.K.; Cai, Y. Engrailed acts with Nejire to control decapentaplegic expression in the Drosophila ovarian stem cell niche. *Development* **2017**, *144*, 3224–3231.
213. Koe, C.T.; Li, S.; Rossi, F.; Wong, J.J.; Wang, Y.; Zhang, Z.; Chen, K.; Aw, S.S.; Richardson, H.E.; Robson, P., et al. The Brm-HDAC3-Erm repressor complex suppresses dedifferentiation in *Drosophila* type II neuroblast lineages. *eLife* **2014**, *3*, e01906.
214. Janssens, D.H.; Komori, H.; Grbac, D.; Chen, K.; Koe, C.T.; Wang, H.; Lee, C.Y. Earmuff restricts progenitor cell potential by attenuating the competence to respond to self-renewal factors. *Development* **2014**, *141*, 1036–1046.
215. Shen, G.; Wu, J.; Han, C.; Liu, H.; Xu, Y.; Zhang, H.; Lin, Y.; Xia, Q. Oestrogen-related receptor reduces vitellogenin expression by crosstalk with the ecdysone receptor pathway in female silkworm, *Bombyx mori*. *Insect Mol. Biol.* **2018**, 10.1111/imb.12385.
216. Jin, W.; Jia, Y.; Tan, E.; Xi, G. Relevance of estrogen-related receptor gene and ecdysone receptor gene in adult testis of the cricket *Teleogryllus emma* (Orthoptera: Gryllidae). *Sci. Nat.* **2017**, *104*, 97.
217. Nakao, H. Analyses of interactions among pair-rule genes and the gap gene *Krüppel* in *Bombyx* segmentation. *Dev. Biol.* **2015**, *405*, 149–157.
218. Jafari, S.; Alkhori, L.; Schleiffer, A.; Brochtrup, A.; Hummel, T.; Alenius, M. Combinatorial activation and repression by seven transcription factors specify *Drosophila* odorant receptor expression. *PLoS Biol.* **2012**, *10*, e1001280.
219. Nagoshi, E.; Sugino, K.; Kula, E.; Okazaki, E.; Tachibana, T.; Nelson, S.; Rosbash, M. Dissecting differential gene expression within the circadian neuronal circuit of *Drosophila*. *Nat. Neurosci.* **2010**, *13*, 60–68.

220. Varma, D.; Bulow, M.H.; Pesch, Y.Y.; Loch, G.; Hoch, M. Forkhead, a new cross regulator of metabolism and innate immunity downstream of TOR in *Drosophila*. *J. Insect Physiol.* **2014**, *69*, 80–88.
221. Zhou, B.; Bagri, A.; Beckendorf, S.K. Salivary gland determination in *Drosophila*: a salivary-specific, *fork head* enhancer integrates spatial pattern and allows *fork head* autoregulation. *Dev. Biol.* **2001**, *237*, 54–67.
222. Abrams, E.W.; Mihoulides, W.K.; Andrew, D.J. Fork head and Sage maintain a uniform and patent salivary gland lumen through regulation of two downstream target genes, *PH4alphaSG1* and *PH4alphaSG2*. *Development* **2006**, *133*, 3517–3527.
223. Fox, R.M.; Vaishnavi, A.; Maruyama, R.; Andrew, D.J. Organ-specific gene expression: the bHLH protein Sage provides tissue specificity to *Drosophila* FoxA. *Development* **2013**, *140*, 2160–2171.
224. Myat, M.M.; Isaac, D.D.; Andrew, D.J. Early genes required for salivary gland fate determination and morphogenesis in *Drosophila melanogaster*. *Adv. Dent. Res.* **2000**, *14*, 89–98.
225. Chandrasekaran, V.; Beckendorf, S.K. *senseless* is necessary for the survival of embryonic salivary glands in *Drosophila*. *Development* **2003**, *130*, 4719–4728.
226. Lehmann, M.; Korge, G. The *fork head* product directly specifies the tissue-specific hormone responsiveness of the *Drosophila Sgs-4* gene. *EMBO J.* **1996**, *15*, 4825–4834.
227. Renault, N.; King-Jones, K.; Lehmann, M. Downregulation of the tissue-specific transcription factor Fork head by *Broad-Complex* mediates a stage-specific hormone response. *Development* **2001**, *128*, 3729–3737.
228. Liu, Y.; Lehmann, M. Genes and biological processes controlled by the *Drosophila* FOXA orthologue Fork head. *Insect Mol. Biol.* **2008**, *17*, 91–101.
229. Bülow, M.H.; Aebersold, R.; Pankratz, M.J.; Jünger, M.A. The *Drosophila* FoxA ortholog Fork head regulates growth and gene expression downstream of Target of rapamycin. *PLoS One* **2010**, *5*, e15171.
230. Zhao, X.; Liu, C.; Li, Q.; Hu, W.; Zhou, M.; Nie, H.; Zhang, Y.; Peng, Z.; Zhao, P.; Xia, Q. Basic helix-loop-helix transcription factor Bmsage is involved in regulation of *fibroin H-chain* gene via interaction with SGF1 in *Bombyx mori*. *PLoS One* **2014**, *9*, e94091.
231. Hui, C.C.; Matsuno, K.; Suzuki, Y. Fibroin gene promoter contains a cluster of homeodomain binding sites that interact with three silk gland factors. *J. Mol. Biol.* **1990**, *213*, 651–670.
232. Mach, V.; Takiya, S.; Ohno, K.; Handa, H.; Imai, T.; Suzuki, Y. Silk gland factor-1 involved in the regulation of *Bombyx* sericin-1 gene contains fork head motif. *J. Biol. Chem.* **1995**, *270*, 9340–9346.
233. Julien, E.; Bordeaux, M.C.; Garel, A.; Couble, P. Fork head alternative binding drives stage-specific gene expression in the silk gland of *Bombyx mori*. *Insect Biochem. Mol. Biol.* **2002**, *32*, 377–387.
234. Zhong, X.; Chowdhury, M.; Li, C.F.; Yu, X.Q. Transcription factor forkhead regulates expression of antimicrobial peptides in the tobacco hornworm, *Manduca sexta*. *Sci. Rep.* **2017**, *7*, 2688.
235. Bao, B.; Hong, B.; Feng, Q.L.; Xu, W.H. Transcription factor fork head regulates the promoter of diapause hormone gene in the cotton bollworm, *Helicoverpa armigera*, and the modification of SUMOylation. *Insect Biochem. Mol. Biol.* **2011**, *41*, 670–679.
236. Li, J.; Ma, Y.; Yuan, W.; Xiao, Y.; Liu, C.; Wang, J.; Peng, J.; Peng, R.; Soberón, M.; Bravo, A., et al. FOXA transcriptional factor modulates insect susceptibility to *Bacillus thuringiensis* Cry1Ac toxin by regulating the expression of toxin-receptor *ABCC2* and *ABCC3* genes. *Insect Biochem. Mol. Biol.* **2017**, *88*, 1–11.
237. Hanlon, C.D.; Andrew, D.J. *Drosophila* FoxL1 non-autonomously coordinates organ placement during embryonic development. *Dev. Biol.* **2016**, *419*, 273–284.
238. Ye, Y.X.; Pan, P.L.; Xu, J.Y.; Shen, Z.F.; Kang, D.; Lu, J.B.; Hu, Q.L.; Huang, H.J.; Lou, Y.H.; Zhou, N.M., et al. Forkhead box transcription factor L2 activates *Fcp3C* to regulate insect chorion formation. *Open Biol.* **2017**, *7*, 170061.
239. Vihervaara, T.; Puig, O. dFOXO regulates transcription of a *Drosophila* acid lipase. *J. Mol. Biol.* **2008**, *376*, 1215–1223.
240. Kang, P.; Chang, K.; Liu, Y.; Bouska, M.; Birnbaum, A.; Karashchuk, G.; Thakore, R.; Zheng, W.; Post, S.; Brent, C.S., et al. *Drosophila* Kruppel homolog 1 represses lipolysis through interaction with dFOXO. *Sci. Rep.* **2017**, *7*, 16369.

241. Becker, T.; Loch, G.; Beyer, M.; Zinke, I.; Aschenbrenner, A.C.; Carrera, P.; Inhester, T.; Schultze, J.L.; Hoch, M. FOXO-dependent regulation of innate immune homeostasis. *Nature* **2010**, *463*, 369–373.
242. Koyama, T.; Rodrigues, M.A.; Athanasiadis, A.; Shingleton, A.W.; Mirth, C.K. Nutritional control of body size through FoxO-Ultraspiracle mediated ecdysone biosynthesis. *eLife* **2014**, *3*, e03091.
243. Hossain, M.S.; Liu, Y.; Zhou, S.; Li, K.; Tian, L.; Li, S. 20-Hydroxyecdysone-induced transcriptional activity of FoxO upregulates *brummer* and *acid lipase-1* and promotes lipolysis in *Bombyx* fat body. *Insect Biochem. Mol. Biol.* **2013**, *43*, 829–838.
244. Zeng, B.; Huang, Y.; Xu, J.; Shiotsuki, T.; Bai, H.; Palli, S.R.; Huang, Y.; Tan, A. The FOXO transcription factor controls insect growth and development by regulating juvenile hormone degradation in the silkworm, *Bombyx mori*. *J. Biol. Chem.* **2017**, *292*, 11659–11669.
245. Baumann, A.A.; Benoit, J.B.; Michalkova, V.; Mireji, P.; Attardo, G.M.; Moulton, J.K.; Wilson, T.G.; Aksoy, S. Juvenile hormone and insulin suppress lipolysis between periods of lactation during tsetse fly pregnancy. *Mol. Cell. Endocrinol.* **2013**, *372*, 30–41.
246. Borras-Castells, F.; Nieva, C.; Maestro, J.L.; Maestro, O.; Belles, X.; Martin, D. Juvenile hormone biosynthesis in adult *Blattella germanica* requires nuclear receptors Seven-up and FTZ-F1. *Sci. Rep.* **2017**, *7*, 40234.
247. Dubrovsky, E.B.; Dubrovskaya, V.A.; Bernardo, T.; Otte, V.; DiFilippo, R.; Bryan, H. The *Drosophila* FTZ-F1 nuclear receptor mediates juvenile hormone activation of *E75A* gene expression through an intracellular pathway. *J. Biol. Chem.* **2011**, *286*, 33689–33700.
248. Parvy, J.P.; Blais, C.; Bernard, F.; Warren, J.T.; Petryk, A.; Gilbert, L.I.; O'Connor, M.B.; Dauphin-Villemant, C. A role for βFTZ-F1 in regulating ecdysteroid titers during post-embryonic development in *Drosophila melanogaster*. *Dev. Biol.* **2005**, *282*, 84–94.
249. Parvy, J.P.; Wang, P.; Garrido, D.; Maria, A.; Blais, C.; Poidevin, M.; Montagne, J. Forward and feedback regulation of cyclic steroid production in *Drosophila melanogaster*. *Development* **2014**, *141*, 3955–3965.
250. Murata, T.; Kageyama, Y.; Hirose, S.; Ueda, H. Regulation of the *EDG84A* gene by FTZ-F1 during metamorphosis in *Drosophila melanogaster*. *Mol. Cell. Biol.* **1996**, *16*, 6509–6515.
251. Kawasaki, H.; Hirose, S.; Ueda, H. βFTZ-F1 dependent and independent activation of *Edg78E*, a pupal cuticle gene, during the early metamorphic period in *Drosophila melanogaster*. *Dev. Growth Differ.* **2002**, *44*, 419–425.
252. Akagi, K.; Kageyama, Y.; Kayashima, Y.; Takakura, Y.; Hirose, S.; Ueda, H. The binding of multiple nuclear receptors to a single regulatory region is important for the proper expression of *EDG84A* in *Drosophila melanogaster*. *J. Mol. Biol.* **2013**, *425*, 71–81.
253. Bond, N.D.; Nelliot, A.; Bernardo, M.K.; Ayerh, M.A.; Gorski, K.A.; Hoshizaki, D.K.; Woodard, C.T. ßFTZ-F1 and Matrix metalloproteinase 2 are required for fat-body remodeling in *Drosophila*. *Dev. Biol.* **2011**, *360*, 286–296.
254. Deng, H.; Zheng, S.; Yang, X.; Liu, L.; Feng, Q. Transcription factors BmPOUM2 and BmβFTZ-F1 are involved in regulation of the expression of the wing cuticle protein gene *BmWCP4* in the silkworm, *Bombyx mori*. *Insect Mol. Biol.* **2011**, *20*, 45–60.
255. Shiomi, K.; Niimi, T.; Imai, K.; Yamashita, O. Structure of the VAP-peptide (*BmA*CP-6.7) gene in the silkworm, *Bombyx mori* and a possible regulation of its expression by BmFTZ-F1. *Insect Biochem. Mol. Biol.* **2000**, *30*, 119–125.
256. Zhou, C.Y.; Zha, X.F.; Shi, P.P.; Zhao, P.; Wang, H.; Zheng, R.W.; Xia, Q.Y. Nuclear hormone receptor BmFTZ-F1 is involved in regulating the *fibroin heavy chain* gene in the silkworm, *Bombyx mori*. *Biochim. Biophys. Acta* **2016**, *1860*, 2529–2536.
257. Vyazunova, I.; Lan, Q. *Yellow fever mosquito sterol carrier protein-2* gene structure and transcriptional regulation. *Insect Mol. Biol.* **2010**, *19*, 205–215.
258. Swantek, D.; Gergen, J.P. Ftz modulates Runt-dependent activation and repression of segment-polarity gene transcription. *Development* **2004**, *131*, 2281–2290.
259. Hang, S.; Gergen, J.P. Different modes of enhancer-specific regulation by Runt and Even-skipped during *Drosophila* segmentation. *Mol. Biol. Cell* **2017**, *28*, 681–691.
260. Sourmeli, S.; Kravariti, L.; Lecanidou, R. In vitro analysis of *Bombyx mori* early chorion gene

regulation: stage specific expression involves interactions with C/EBP-like and GATA factors. *Insect Biochem. Mol. Biol.* **2003**, *33*, 525–540.
261. Papantonis, A.; Vanden Broeck, J.; Lecanidou, R. Architectural factor HMGA induces promoter bending and recruits C/EBP and GATA during silkmoth chorion gene regulation. *Biochem. J.* **2008**, *416*, 85–97.
262. Rao, X.J.; Xu, X.X.; Yu, X.Q. *Manduca sexta* moricin promoter elements can increase promoter activities of *Drosophila melanogaster* antimicrobial peptide genes. *Insect Biochem. Mol. Biol.* **2011**, *41*, 982–992.
263. Godlewski, J.; Wang, S.; Wilson, T.G. Interaction of bHLH-PAS proteins involved in juvenile hormone reception in *Drosophila*. *Biochem. Biophys. Res. Commun.* **2006**, *342*, 1305–1311.
264. Janssens, H.; Hou, S.; Jaeger, J.; Kim, A.R.; Myasnikova, E.; Sharp, D.; Reinitz, J. Quantitative and predictive model of transcriptional control of the *Drosophila melanogaster even skipped* gene. *Nat. Genet.* **2006**, *38*, 1159–1165.
265. Han, Z.; Yi, P.; Li, X.; Olson, E.N. Hand, an evolutionarily conserved bHLH transcription factor required for *Drosophila* cardiogenesis and hematopoiesis. *Development* **2006**, *133*, 1175–1182.
266. Popichenko, D.; Sellin, J.; Bartkuhn, M.; Paululat, A. Hand is a direct target of the forkhead transcription factor Biniou during *Drosophila* visceral mesoderm differentiation. *BMC Dev. Biol.* **2007**, *7*, 49.
267. Tögel, M.; Meyer, H.; Lehmacher, C.; Heinisch, J.J.; Pass, G.; Paululat, A. The bHLH transcription factor hand is required for proper wing heart formation in *Drosophila*. *Dev. Biol.* **2013**, *381*, 446–459.
268. Hallier, B.; Hoffmann, J.; Roeder, T.; Tögel, M.; Meyer, H.; Paululat, A. The bHLH transcription factor Hand regulates the expression of genes critical to heart and muscle function in *Drosophila melanogaster*. *PLoS One* **2015**, *10*, e0134204.
269. Saha, T.T.; Shin, S.W.; Dou, W.; Roy, S.; Zhao, B.; Hou, Y.; Wang, X.L.; Zou, Z.; Girke, T.; Raikhel, A.S. Hairy and Groucho mediate the action of juvenile hormone receptor Methoprene-tolerant in gene repression. *Proc. Natl. Acad. Sci. U. S. A.* **2016**, *113*, E735–E743.
270. Nakao, H. Hunchback knockdown induces supernumerary segment formation in *Bombyx*. *Dev. Biol.* **2016**, *413*, 207–216.
271. Isshiki, T.; Pearson, B.; Holbrook, S.; Doe, C.Q. *Drosophila* neuroblasts sequentially express transcription factors which specify the temporal identity of their neuronal progeny. *Cell* **2001**, *106*, 511–521.
272. Torres-Oliva, M.; Schneider, J.; Wiegleb, G.; Kaufholz, F.; Posnien, N. Dynamic genome wide expression profiling of *Drosophila* head development reveals a novel role of Hunchback in retinal glia cell development and blood-brain barrier integrity. *PLoS Genet.* **2018**, *14*, e1007180.
273. Wang, X.; Hou, Y.; Saha, T.T.; Pei, G.; Raikhel, A.S.; Zou, Z. Hormone and receptor interplay in the regulation of mosquito lipid metabolism. *Proc. Natl. Acad. Sci. U. S. A.* **2017**, *114*, E2709–E2718.
274. Georgias, C.; Wasser, M.; Hinz, U. A basic-helix-loop-helix protein expressed in precursors of *Drosophila* longitudinal visceral muscles. *Mech. Dev.* **1997**, *69*, 115–124.
275. Shukla, V.; Habib, F.; Kulkarni, A.; Ratnaparkhi, G.S. Gene duplication, lineage-specific expansion, and subfunctionalization in the MADF-BESS family patterns the *Drosophila* wing hinge. *Genetics* **2014**, *196*, 481–496.
276. Lam, G.; Hall, B.L.; Bender, M.; Thummel, C.S. *DHR3* is required for the prepupal-pupal transition and differentiation of adult structures during *Drosophila* metamorphosis. *Dev. Biol.* **1999**, *212*, 204–216.
277. Ou, Q.; Magico, A.; King-Jones, K. Nuclear receptor DHR4 controls the timing of steroid hormone pulses during *Drosophila* development. *PLoS Biol.* **2011**, *9*, e1001160.
278. Gu, S.H.; Hsieh, Y.C.; Lin, P.L. Stimulation of orphan nuclear receptor HR38 gene expression by PTTH in prothoracic glands of the silkworm, *Bombyx mori*. *J. Insect Physiol.* **2016**, *90*, 8–16.
279. King-Jones, K.; Horner, M.A.; Lam, G.; Thummel, C.S. The DHR96 nuclear receptor regulates xenobiotic responses in *Drosophila*. *Cell Metab.* **2006**, *4*, 37–48.
280. Westwood, J.T.; Clos, J.; Wu, C. Stress-induced oligomerization and chromosomal relocalization of heat-shock factor. *Nature* **1991**, *353*, 822–827.
281. Guertin, M.J.; Petesch, S.J.; Zobeck, K.L.; Min, I.M.; Lis, J.T. *Drosophila* heat shock system as a

general model to investigate transcriptional regulation. *Cold Spring Harb. Symp. Quant. Biol.* **2010**, *75*, 1–9.
282. Kihara, F.; Niimi, T.; Yamashita, O.; Yaginuma, T. Heat shock factor binds to heat shock elements upstream of heat shock protein 70a and Samui genes to confer transcriptional activity in *Bombyx mori* diapause eggs exposed to 5 degrees C. *Insect Biochem. Mol. Biol.* **2011**, *41*, 843–851.
283. Azpiazu, N.; Morata, G. Function and regulation of *homothorax* in the wing imaginal disc of *Drosophila*. *Development* **2000**, *127*, 2685–2693.
284. Casares, F.; Mann, R.S. A dual role for *homothorax* in inhibiting wing blade development and specifying proximal wing identities in *Drosophila*. *Development* **2000**, *127*, 1499–1508.
285. Niu, K.; Zhang, X.; Deng, H.; Wu, F.; Ren, Y.; Xiang, H.; Zheng, S.; Liu, L.; Huang, L.; Zeng, B., et al. BmILF and i-motif structure are involved in transcriptional regulation of *BmPOUM2* in *Bombyx mori*. *Nucleic Acids Res.* **2018**, *46*, 1710–1723.
286. Danielsen, E.T.; Moeller, M.E.; Dorry, E.; Komura-Kawa, T.; Fujimoto, Y.; Troelsen, J.T.; Herder, R.; O'Connor, M.B.; Niwa, R.; Rewitz, K.F. Transcriptional control of steroid biosynthesis genes in the *Drosophila* prothoracic gland by ventral veins lacking and knirps. *PLoS Genet.* **2014**, *10*, e1004343.
287. Mishra, A.K.; Bargmann, B.O.R.; Tsachaki, M.; Fritsch, C.; Sprecher, S.G. Functional genomics identifies regulators of the phototransduction machinery in the *Drosophila* larval eye and adult ocelli. *Dev. Biol.* **2016**, *410*, 164–177.
288. Kayukawa, T.; Nagamine, K.; Ito, Y.; Nishita, Y.; Ishikawa, Y.; Shinoda, T. Krüppel homolog 1 inhibits insect metamorphosis via direct transcriptional repression of *Broad-Complex*, a pupal specifier gene. *J. Biol. Chem.* **2016**, *291*, 1751–1762.
289. Kayukawa, T.; Jouraku, A.; Ito, Y.; Shinoda, T. Molecular mechanism underlying juvenile hormone-mediated repression of precocious larval-adult metamorphosis. *Proc. Natl. Acad. Sci. U. S. A.* **2017**, *114*, 1057–1062.
290. Belles, X.; Santos, C.G. The MEKRE93 (Methoprene tolerant-Krüppel homolog 1-E93) pathway in the regulation of insect metamorphosis, and the homology of the pupal stage. *Insect Biochem. Mol. Biol.* **2014**, *52*, 60–68.
291. Zhang, T.; Song, W.; Li, Z.; Qian, W.; Wei, L.; Yang, Y.; Wang, W.; Zhou, X.; Meng, M.; Peng, J., et al. Krüppel homolog 1 represses insect ecdysone biosynthesis by directly inhibiting the transcription of steroidogenic enzymes. *Proc. Natl. Acad. Sci. U. S. A.* **2018**, *115*, 3960–3965.
292. Song, J.; Wu, Z.; Wang, Z.; Deng, S.; Zhou, S. Krüppel-homolog 1 mediates juvenile hormone action to promote vitellogenesis and oocyte maturation in the migratory locust. *Insect Biochem. Mol. Biol.* **2014**, *52*, 94–101.
293. Urñna, E.; Chafino, S.; Manjón, C.; Franch-Marro, X.; Martín, D. The occurrence of the holometabolous pupal stage requires the interaction between E93, Krüppel-Homolog 1 and Broad-Complex. *PLoS Genet.* **2016**, *12*, e1006020.
294. Smykal, V.; Bajgar, A.; Provaznik, J.; Fexova, S.; Buricova, M.; Takaki, K.; Hodkova, M.; Jindra, M.; Dolezel, D. Juvenile hormone signaling during reproduction and development of the linden bug, *Pyrrhocoris apterus*. *Insect Biochem. Mol. Biol.* **2014**, *45*, 69–76.
295. Smith, R.C.; Eappen, A.G.; Radtke, A.J.; Jacobs-Lorena, M. Regulation of anti-*Plasmodium* immunity by a *LITAF*-like transcription factor in the malaria vector *Anopheles gambiae*. *PLoS Pathog.* **2012**, *8*, e1002965.
296. Carmena, A.; Bate, M.; Jiménez, F. *lethal of scute*, a proneural gene, participates in the specification of muscle progenitors during *Drosophila* embryogenesis. *Genes Dev.* **1995**, *9*, 2373–2383.
297. Kerman, B.E.; Cheshire, A.M.; Myat, M.M.; Andrew, D.J. Ribbon modulates apical membrane during tube elongation through Crumbs and Moesin. *Dev. Biol.* **2008**, *320*, 278–288.
298. Certel, S.J.; Thor, S. Specification of *Drosophila* motoneuron identity by the combinatorial action of POU and LIM-HD factors. *Development* **2004**, *131*, 5429–5439.
299. Steiger, D.; Furrer, M.; Schwinkendorf, D.; Gallant, P. Max-independent functions of Myc in *Drosophila melanogaster*. *Nat. Genet.* **2008**, *40*, 1084–1091.
300. Kayukawa, T.; Minakuchi, C.; Namiki, T.; Togawa, T.; Yoshiyama, M.; Kamimura, M.; Mita, K.; Imanishi, S.; Kiuchi, M.; Ishikawa, Y., et al. Transcriptional regulation of juvenile hormone-mediated induction of Krüppel homolog 1, a repressor of insect metamorphosis. *Proc.*

*Natl. Acad. Sci. U. S. A.* **2012**, *109*, 11729–11734.
301. Kayukawa, T.; Shinoda, T. Functional characterization of two paralogous JH receptors, methoprene-tolerant 1 and 2, in the silkworm, *Bombyx mori* (Lepidoptera: Bombycidae). *Appl. Entomol. Zool.* **2015**, *50*, 383–391.
302. Gujar, H.; Palli, S.R. Juvenile hormone regulation of female reproduction in the common bed bug, *Cimex lectularius*. *Sci. Rep.* **2016**, *6*, 35546.
303. Liu, Y.; Sheng, Z.; Liu, H.; Wen, D.; He, Q.; Wang, S.; Shao, W.; Jiang, R.J.; An, S.; Sun, Y., et al. Juvenile hormone counteracts the bHLH-PAS transcription factors MET and GCE to prevent caspase-dependent programmed cell death in *Drosophila*. *Development* **2009**, *136*, 2015–2025.
304. Meng, Q.W.; Xu, Q.Y.; Deng, P.; Fu, K.Y.; Guo, W.C.; Li, G.Q. Transcriptional response of Methoprene-tolerant (Met) gene to three insect growth disruptors in *Leptinotarsa decemlineata* (Say). *J. Asia Pac. Entomol.* **2018**, *21*, 466–473.
305. Meng, Q.W.; Xu, Q.Y.; Deng, P.; Fu, K.Y.; Guo, W.C.; Li, G.Q. Involvement of methoprene-tolerant (Met) in the determination of the final body size in *Leptinotarsa decemlineata* (Say) larvae. *Insect Biochem. Mol. Biol.* **2018**, *97*, 1–9.
306. Guo, W.; Wu, Z.; Song, J.; Jiang, F.; Wang, Z.; Deng, S.; Walker, V.K.; Zhou, S. Juvenile hormone-receptor complex acts on *Mcm4* and *Mcm7* to promote polyploidy and vitellogenesis in the migratory locust. *PLoS Genet.* **2014**, *10*, e1004702.
307. Wu, Z.; Guo, W.; Xie, Y.; Zhou, S. Juvenile hormone activates the transcription of cell-division-cycle 6 (*Cdc6*) for polyploidy-dependent insect vitellogenesis and oogenesis. *J. Biol. Chem.* **2016**, *291*, 5418–5427.
308. Luo, M.; Li, D.; Wang, Z.; Guo, W.; Kang, L.; Zhou, S. Juvenile hormone differentially regulates two Grp78 genes encoding protein chaperones required for insect fat body cell homeostasis and vitellogenesis. *J. Biol. Chem.* **2017**, *292*, 8823–8834.
309. Hejnikova, M.; Paroulek, M.; Hodkova, M. Decrease in *Methoprene tolerant* and *Taiman* expression reduces juvenile hormone effects and enhances the levels of juvenile hormone circulating in males of the linden bug *Pyrrhocoris apterus*. *J. Insect Physiol.* **2016**, *93-94*, 72–80.
310. Konopova, B.; Jindra, M. Juvenile hormone resistance gene Methoprene-tolerant controls entry into metamorphosis in the beetle *Tribolium castaneum*. *Proc. Natl. Acad. Sci. U. S. A.* **2007**, *104*, 10488–10493.
311. Parthasarathy, R.; Tan, A.; Palli, S.R. bHLH-PAS family transcription factor methoprene-tolerant plays a key role in JH action in preventing the premature development of adult structures during larval-pupal metamorphosis. *Mech. Dev.* **2008**, *125*, 601–616.
312. Minakuchi, C.; Namiki, T.; Shinoda, T. *Krüppel homolog 1*, an early juvenile hormone-response gene downstream of *Methoprene-tolerant*, mediates its anti-metamorphic action in the red flour beetle *Tribolium castaneum*. *Dev. Biol.* **2009**, *325*, 341–350.
313. Charles, J.P.; Iwema, T.; Epa, V.C.; Takaki, K.; Rynes, J.; Jindra, M. Ligand-binding properties of a juvenile hormone receptor, Methoprene-tolerant. *Proc. Natl. Acad. Sci. U. S. A.* **2011**, *108*, 21128–21133.
314. Kayukawa, T.; Tateishi, K.; Shinoda, T. Establishment of a versatile cell line for juvenile hormone signaling analysis in *Tribolium castaneum*. *Sci. Rep.* **2013**, *3*, 1570.
315. Zhu, J.; Busche, J.M.; Zhang, X. Identification of juvenile hormone target genes in the adult female mosquitoes. *Insect Biochem. Mol. Biol.* **2010**, *40*, 23–29.
316. Li, M.; Mead, E.A.; Zhu, J. Heterodimer of two bHLH-PAS proteins mediates juvenile hormone-induced gene expression. *Proc. Natl. Acad. Sci. U. S. A.* **2011**, *108*, 638–643.
317. Zou, Z.; Saha, T.T.; Roy, S.; Shin, S.W.; Backman, T.W.; Girke, T.; White, K.P.; Raikhel, A.S. Juvenile hormone and its receptor, methoprene-tolerant, control the dynamics of mosquito gene expression. *Proc. Natl. Acad. Sci. U. S. A.* **2013**, *110*, E2173–E2181.
318. Cui, Y.; Sui, Y.; Xu, J.; Zhu, F.; Palli, S.R. Juvenile hormone regulates *Aedes aegypti* Krüppel homolog 1 through a conserved E box motif. *Insect Biochem. Mol. Biol.* **2014**, *52*, 23–32.
319. Shin, S.W.; Zou, Z.; Saha, T.T.; Raikhel, A.S. bHLH-PAS heterodimer of Methoprene-tolerant and Cycle mediates circadian expression of juvenile hormone-induced mosquito genes. *Proc. Natl. Acad. Sci. U. S. A.* **2012**, *109*, 16576–16581.

320. Takiya, S.; Inoue, H.; Kimoto, M. Novel enhancer and promoter elements indispensable for the tissue-specific expression of the *sericin-1* gene of the silkworm *Bombyx mori*. *Insect Biochem. Mol. Biol.* **2011**, *41*, 592–601.
321. Kimoto, M.; Kitagawa, T.; Kobayashi, I.; Nakata, T.; Kuroiwa, A.; Takiya, S. Inhibition of the binding of MSG-intermolt-specific complex, MIC, to the *sericin-1* gene promoter and *sericin-1* gene expression by POU-M1/SGF-3. *Dev. Genes Evol.* **2012**, *222*, 351–359.
322. Kimoto, M.; Tsubota, T.; Uchino, K.; Sezutsu, H.; Takiya, S. Hox transcription factor Antp regulates *sericin-1* gene expression in the terminal differentiated silk gland of *Bombyx mori*. *Dev. Biol.* **2014**, *386*, 64–71.
323. Fu, C.L.; Wang, X.F.; Cheng, Q.; Wang, D.; Hirose, S.; Liu, Q.X. The T-box transcription factor Midline regulates wing development by repressing *wingless* and *hedgehog* in *Drosophila*. *Sci. Rep.* **2016**, *6*, 27981.
324. Gaziova, I.; Bhat, K.M. Ancestry-independent fate specification and plasticity in the developmental timing of a typical *Drosophila* neuronal lineage. *Development* **2009**, *136*, 263–274.
325. Hallsson, J.H.; Haflidadóttir, B.S.; Stivers, C.; Odenwald, W.; Arnheiter, H.; Pignoni, F.; Steingrimsson, E. The basic helix-loop-helix leucine zipper transcription factor Mitf is conserved in *Drosophila* and functions in eye development. *Genetics* **2004**, *167*, 233–241.
326. Bouche, V.; Espinosa, A.P.; Leone, L.; Sardiello, M.; Ballabio, A.; Botas, J. *Drosophila* Mitf regulates the V-ATPase and the lysosomal-autophagic pathway. *Autophagy* **2016**, *12*, 484–498.
327. Neubueser, D.; Warren, J.T.; Gilbert, L.I.; Cohen, S.M. *molting defective* is required for ecdysone biosynthesis. *Dev. Biol.* **2005**, *280*, 362–372.
328. Ono, H.; Rewitz, K.F.; Shinoda, T.; Itoyama, K.; Petryk, A.; Rybczynski, R.; Jarcho, M.; Warren, J.T.; Marqués, G.; Shimell, M.J., et al. *Spook* and *Spookier* code for stage-specific components of the ecdysone biosynthetic pathway in *Diptera*. *Dev. Biol.* **2006**, *298*, 555–570.
329. Uryu, O.; Ou, Q.; Komura-Kawa, T.; Kamiyama, T.; Iga, M.; Syrzycka, M.; Hirota, K.; Kataoka, H.; Honda, B.M.; King-Jones, K., et al. Cooperative control of ecdysone biosynthesis in *Drosophila* by transcription factors Séance, Ouija board, and Molting defective. *Genetics* **2017**, *208*, 605–622.
330. Havula, E.; Teesalu, M.; Hyötyläinen, T.; Seppälä, H.; Hasygar, K.; Auvinen, P.; Orešič, M.; Sandmann, T.; Hietakangas, V. Mondo/ChREBP-Mlx-regulated transcriptional network is essential for dietary sugar tolerance in *Drosophila*. *PLoS Genet.* **2013**, *9*, e1003438.
331. Loo, L.W.; Secombe, J.; Little, J.T.; Carlos, L.S.; Yost, C.; Cheng, P.F.; Flynn, E.M.; Edgar, B.A.; Eisenman, R.N. The transcriptional repressor dMnt is a regulator of growth in *Drosophila melanogaster*. *Mol. Cell Biol.* **2005**, *25*, 7078–7091.
332. Paterson, B.M.; Shirakata, M.; Nakamura, S.; Dechesne, C.; Walldorf, U.; Eldridge, J.; Dubendorfer, A.; Frasch, M.; Gehring, W.J. Isolation and functional comparison of Dmyd, the *Drosophila* homologue of the vertebrate myogenic determination genes, with CMD1. *Symp. Soc. Exp. Biol.* **1992**, *46*, 89–109.
333. Schmidt, I.; Franzdóttir, S.R.; Edenfeld, G.; Rodrigues, F.; Zierau, A.; Klämbt, C. Transcriptional regulation of peripheral glial cell differentiation in the embryonic nervous system of *Drosophila*. *Glia* **2011**, *59*, 1264–1272.
334. Aufauvre, J.; Misme-Aucouturier, B.; Viguès, B.; Texier, C.; Delbac, F.; Blot, N. Transcriptome analyses of the honeybee response to *Nosema ceranae* and insecticides. *PLoS One* **2014**, *9*, e91686.
335. Braid, L.R.; Lee, W.; Uetrecht, A.C.; Swarup, S.; Papaianni, G.; Heiler, A.; Verheyen, E.M. Nemo phosphorylates Even-skipped and promotes Eve-mediated repression of *odd-skipped* in even parasegments during *Drosophila* embryogenesis. *Dev. Biol.* **2010**, *343*, 178–189.
336. Oyallon, J.; Apitz, H.; Miguel-Aliaga, I.; Timofeev, K.; Ferreira, L.; Salecker, I. Regulation of locomotion and motoneuron trajectory selection and targeting by the *Drosophila* homolog of Olig family transcription factors. *Dev. Biol.* **2012**, *369*, 261–276.
337. Chen, D.; Gu, T.; Pham, T.N.; Zachary, M.J.; Hewes, R.S. Regulatory mechanisms of metamorphic neuronal remodeling revealed through a genome-wide modifier screen in *Drosophila melanogaster*. *Genetics* **2017**, *206*, 1429–1443.
338. Zattara, E.E.; Macagno, A.L.M.; Busey, H.A.; Moczek, A.P. Development of functional ectopic compound eyes in scarabaeid beetles by knockdown of *orthodenticle*. *Proc. Natl. Acad. Sci. U. S. A.*

**2017**, *114*, 12021–12026.
339. Heffer, A.; Pick, L. Conservation and variation in Hox genes: how insect models pioneered the evo-devo field. *Annu. Rev. Entomol.* **2013**, *58*, 161–179.
340. Junell, A.; Uvell, H.; Pick, L.; Engström, Y. Isolation of regulators of *Drosophila* immune defense genes by a double interaction screen in yeast. *Insect Biochem. Mol. Biol.* **2007**, *37*, 202–212.
341. Lin, S.C.; Lin, M.H.; Horváth, P.; Reddy, K.L.; Storti, R.V. PDP1, a novel *Drosophila* PAR domain bZIP transcription factor expressed in developing mesoderm, endoderm and ectoderm, is a transcriptional regulator of somatic muscle genes. *Development* **1997**, *124*, 4685–4696.
342. Dzitoyeva, S.; Manev, H. Reduction of cellular lipid content by a knockdown of *Drosophila PDP1γ* and mammalian hepatic leukemia factor. *J. Lipids* **2013**, *2013*, 297932.
343. Shiomi, K.; Fujiwara, Y.; Yasukochi, Y.; Kajiura, Z.; Nakagaki, M.; Yaginuma, T. The Pitx homeobox gene in *Bombyx mori*: regulation of DH-PBAN neuropeptide hormone gene expression. *Mol. Cell. Neurosci.* **2007**, *34*, 209–218.
344. Ohtsuka, K.; Atsumi, T.; Fukushima, Y.; Shiomi, K. Identification of a cis-regulatory element that directs prothoracicotropic hormone gene expression in the silkworm *Bombyx mori*. *Insect Biochem. Mol. Biol.* **2011**, *41*, 356–361.
345. Zhu, S.; Barshow, S.; Wildonger, J.; Jan, L.Y.; Jan, Y.N. Ets transcription factor Pointed promotes the generation of intermediate neural progenitors in *Drosophila* larval brains. *Proc. Natl. Acad. Sci. U. S. A.* **2011**, *108*, 20615–20620.
346. Xie, Y.; Li, X.; Deng, X.; Hou, Y.; O'Hara, K.; Urso, A.; Peng, Y.; Chen, L.; Zhu, S. The Ets protein Pointed prevents both premature differentiation and dedifferentiation of *Drosophila* intermediate neural progenitors. *Development* **2016**, *143*, 3109–3118.
347. Fukuta, M.; Matsuno, K.; Hui, C.C.; Nagata, T.; Takiya, S.; Xu, P.X.; Ueno, K.; Suzuki, Y. Molecular cloning of a POU domain-containing factor involved in the regulation of the *Bombyx sericin-1* gene. *J. Biol. Chem.* **1993**, *268*, 19471–19475.
348. Sharma, V.; Pandey, A.K.; Kumar, A.; Misra, S.; Gupta, H.P.K.; Gupta, S.; Singh, A.; Buehner, N.A.; Ravi Ram, K. Functional male accessory glands and fertility in *Drosophila* require novel ecdysone receptor. *PLoS Genet.* **2017**, *13*, e1006788.
349. Zhao, J.; Liu, X.N.; Li, F.; Zhuang, S.Z.; Huang, L.N.; Ma, J.; Gao, X.W. Yeast one-hybrid screening the potential regulator of CYP6B6 overexpression of *Helicoverpa armigera* under 2-tridecanone stress. *Bull. Entomol. Res.* **2016**, *106*, 182–190.
350. Szuplewski, S.; Kottler, B.; Terracol, R. The *Drosophila* bZIP transcription factor Vrille is involved in hair and cell growth. *Development* **2003**, *130*, 3651–3662.
351. Szuplewski, S.; Fraisse-Véron, I.; George, H.; Terracol, R. *vrille* is required to ensure tracheal integrity in *Drosophila* embryo. *Dev. Growth Differ.* **2010**, *52*, 409–418.
352. Monnier, V.; Iché-Torres, M.; Rera, M.; Contremoulins, V.; Guichard, C.; Lalevée, N.; Tricoire, H.; Perrin, L. dJun and Vri/dNFIL3 are major regulators of cardiac aging in *Drosophila*. *PLoS Genet.* **2012**, *8*, e1003081.
353. Gunawardhana, K.L.; Hardin, P.E. VRILLE controls PDF neuropeptide accumulation and arborization rhythms in small ventrolateral neurons to drive rhythmic behavior in *Drosophila*. *Curr. Biol.* **2017**, *27*, 3442–3453.
354. Hanniger, S.; Dumas, P.; Schofl, G.; Gebauer-Jung, S.; Vogel, H.; Unbehend, M.; Heckel, D.G.; Groot, A.T. Genetic basis of allochronic differentiation in the fall armyworm. *BMC Evol. Biol.* **2017**, *17*, 68.
355. Liu, L.; Li, Y.; Wang, Y.; Zhao, P.; Wei, S.; Li, Z.; Chang, H.; He, H. Biochemical characterization and functional analysis of the POU transcription factor POU-M2 of *Bombyx mori*. *Int. J. Biol. Macromol.* **2016**, *86*, 701–708.
356. Meng, M.; Cheng, D.J.; Peng, J.; Qian, W.L.; Li, J.R.; Dai, D.D.; Zhang, T.L.; Xia, Q.Y. The homeodomain transcription factors antennapedia and POU-M2 regulate the transcription of the steroidogenic enzyme gene *Phantom* in the silkworm. *J. Biol. Chem.* **2015**, *290*, 24438–24452.
357. Zhang, T.Y.; Kang, L.; Zhang, Z.F.; Xu, W.H. Identification of a POU factor involved in regulating the neuron-specific expression of the gene encoding diapause hormone and pheromone biosynthesis-activating neuropeptide in *Bombyx mori*. *Biochem. J.* **2004**, *380*, 255–263.

358. Cheng, C.; Ko, A.; Chaieb, L.; Koyama, T.; Sarwar, P.; Mirth, C.K.; Smith, W.A.; Suzuki, Y. The POU factor Ventral veins lacking/Drifter directs the timing of metamorphosis through ecdysteroid and juvenile hormone signaling. *PLoS Genet.* **2014**, *10*, e1004425.
359. Zhang, T.Y.; Xu, W.H. Identification and characterization of a POU transcription factor in the cotton bollworm, *Helicoverpa armigera*. *BMC Mol. Biol.* **2009**, *10*, 25.
360. Song, Z.; Yang, Y.P.; Xu, W.H. PTEN expression responds to transcription factor POU and regulates p-AKT levels during diapause initiation in the cotton bollworm, *Helicoverpa armigera*. *Insect Biochem. Mol. Biol.* **2018**, *100*, 48–58.
361. Stöven, S.; Ando, I.; Kadalayil, L.; Engström, Y.; Hultmark, D. Activation of the *Drosophila* NF-κB factor Relish by rapid endoproteolytic cleavage. *EMBO Rep.* **2000**, *1*, 347–352.
362. Hetru, C.; Hoffmann, J.A. NF-κB in the immune response of *Drosophila*. *Cold Spring Harb. Perspect. Biol.* **2009**, *1*, a000232.
363. Zhang, Y.; Lu, Y.X.; Liu, J.; Yang, C.; Feng, Q.L.; Xu, W.H. A regulatory pathway, ecdysone-transcription factor Relish-cathepsin L, is involved in insect fat body dissociation. *PLoS Genet.* **2013**, *9*, e1003273.
364. Meister, S.; Kanzok, S.M.; Zheng, X.L.; Luna, C.; Li, T.R.; Hoa, N.T.; Clayton, J.R.; White, K.P.; Kafatos, F.C.; Christophides, G.K., et al. Immune signaling pathways regulating bacterial and malaria parasite infection of the mosquito *Anopheles gambiae*. *Proc. Natl. Acad. Sci. U. S. A.* **2005**, *102*, 11420–11425.
365. Shin, S.W.; Kokoza, V.; Bian, G.; Cheon, H.M.; Kim, Y.J.; Raikhel, A.S. REL1, a homologue of *Drosophila* Dorsal, regulates Toll antifungal immune pathway in the female mosquito *Aedes aegypti*. *J. Biol. Chem.* **2005**, *280*, 16499–16507.
366. Shin, S.W.; Kokoza, V.; Ahmed, A.; Raikhel, A.S. Characterization of three alternatively spliced isoforms of the Rel/NF-kappa B transcription factor Relish from the mosquito *Aedes aegypti*. *Proc. Natl. Acad. Sci. U. S. A.* **2002**, *99*, 9978–9983.
367. Shin, S.W.; Kokoza, V.; Lobkov, I.; Raikhel, A.S. Relish-mediated immune deficiency in the transgenic mosquito *Aedes aegypti*. *Proc. Natl. Acad. Sci. U. S. A.* **2003**, *100*, 2616–2621.
368. Antonova, Y.; Alvarez, K.S.; Kim, Y.J.; Kokoza, V.; Raikhel, A.S. The role of NF-κB factor REL2 in the *Aedes aegypti* immune response. *Insect Biochem. Mol. Biol.* **2009**, *39*, 303–314.
369. Paradkar, P.N.; Duchemin, J.-B.; Voysey, R.; Walker, P.J. Dicer-2-dependent activation of *Culex* Vago occurs via the TRAF-Rel2 signaling pathway. In *PLoS Negl. Trop. Dis.*, 2014; Vol. 8, p e2823.
370. Tanaka, H.; Yamamoto, M.; Moriyama, Y.; Yamao, M.; Furukawa, S.; Sagisaka, A.; Nakazawa, H.; Mori, H.; Yamakawa, M. A novel Rel protein and shortened isoform that differentially regulate antibacterial peptide genes in the silkworm *Bombyx mori*. *Biochim. Biophys. Acta* **2005**, *1730*, 10–21.
371. Tanaka, H.; Matsuki, H.; Furukawa, S.; Sagisaka, A.; Kotani, E.; Mori, H.; Yamakawa, M. Identification and functional analysis of Relish homologs in the silkworm, *Bombyx mori*. *Biochim. Biophys. Acta* **2007**, *1769*, 559–568.
372. Sagisaka, A.; Tanaka, H.; Furukawa, S.; Yamakawa, M. Characterization of a homologue of the Rel/NF-κB transcription factor from a beetle, *Allomyrina dichotoma*. *Biochim. Biophys. Acta* **2004**, *1678*, 85–93.
373. Loganathan, R.; Lee, J.S.; Wells, M.B.; Grevengoed, E.; Slattery, M.; Andrew, D.J. *Ribbon* regulates morphogenesis of the *Drosophila* embryonic salivary gland through transcriptional activation and repression. *Dev. Biol.* **2016**, *409*, 234–250.
374. Kramer, S.G.; Jinks, T.M.; Schedl, P.; Gergen, J.P. Direct activation of *Sex-lethal* transcription by the *Drosophila* Runt protein. *Development* **1999**, *126*, 191–200.
375. Stoehr, A.M.; Walker, J.F.; Monteiro, A. Spalt expression and the development of melanic color patterns in pierid butterflies. *EvoDevo* **2013**, *4*, 6.
376. Futahashi, R.; Shirataki, H.; Narita, T.; Mita, K.; Fujiwara, H. Comprehensive microarray-based analysis for stage-specific larval camouflage pattern-associated genes in the swallowtail butterfly, *Papilio xuthus*. *BMC Biol.* **2012**, *10*, 46.
377. Salz, H.K.; Erickson, J.W. Sex determination in *Drosophila*: The view from the top. *Fly* **2010**, *4*, 60–70.
378. Panzer, S.; Weigel, D.; Beckendorf, S.K. Organogenesis in *Drosophila melanogaster*: embryonic

salivary gland determination is controlled by homeotic and dorsoventral patterning genes. *Development* **1992**, *114*, 49–57.
379. Ryoo, H.D.; Mann, R.S. The control of trunk Hox specificity and activity by Extradenticle. *Genes Dev.* **1999**, *13*, 1704–1716.
380. Henderson, K.D.; Andrew, D.J. Regulation and function of *Scr*, *exd*, and *hth* in the *Drosophila* salivary gland. *Dev. Biol.* **2000**, *217*, 362–374.
381. Papadopoulos, D.K.; Skouloudaki, K.; Adachi, Y.; Samakovlis, C.; Gehring, W.J. Dimer formation via the homeodomain is required for function and specificity of Sex combs reduced in *Drosophila*. *Dev. Biol.* **2012**, *367*, 78–89.
382. Kokubo, H.; Ueno, K.; Amanai, K.; Suzuki, Y. Involvement of the *Bombyx Scr* gene in development of the embryonic silk gland. *Dev. Biol.* **1997**, *186*, 46–57.
383. Ma, L.; Li, K.; Guo, Y.; Sun, X.; Deng, H.; Li, K.; Feng, Q.; Li, S. Ras-Raf-MAPK signaling promotes nuclear localization of FOXA transcription factor SGF1 via Ser91 phosphorylation. *Biochim. Biophys. Acta.* **2018**, *1865*, 560–571.
384. Franks, R.G.; Crews, S.T. Transcriptional activation domains of the *single-minded* bHLH protein are required for CNS midline cell development. *Mech. Dev.* **1994**, *45*, 269–277.
385. Hong, J.W.; Park, K.W.; Levine, M.S. Temporal regulation of *single-minded* target genes in the ventral midline of the *Drosophila* central nervous system. *Dev. Biol.* **2013**, *380*, 335–343.
386. Lavista-Llanos, S.; Centanin, L.; Irisarri, M.; Russo, D.M.; Gleadle, J.M.; Bocca, S.N.; Muzzopappa, M.; Ratcliffe, P.J.; Wappner, P. Control of the hypoxic response in *Drosophila melanogaster* by the basic helix-loop-helix PAS protein similar. *Mol. Cell Biol.* **2002**, *22*, 6842–6853.
387. Mukherjee, T.; Kim, W.S.; Mandal, L.; Banerjee, U. Interaction between Notch and Hif-$\alpha$ in development and survival of *Drosophila* blood cells. *Science* **2011**, *332*, 1210–1213.
388. Erickson, J.W.; Cline, T.W. A bZIP protein, Sisterless-a, collaborates with bHLH transcription factors early in *Drosophila* development to determine sex. *Genes Dev.* **1993**, *7*, 1688–1702.
389. Walker, J.J.; Lee, K.K.; Desai, R.N.; Erickson, J.W. The *Drosophila melanogaster* sex determination gene *sisA* is required in yolk nuclei for midgut formation. *Genetics* **2000**, *155*, 191–202.
390. Möller, A.; Avila, F.W.; Erickson, J.W.; Jäckle, H. *Drosophila* BAP60 is an essential component of the Brahma complex, required for gene activation and repression. *J. Mol. Biol.* **2005**, *352*, 329–337.
391. Ashraf, S.I.; Hu, X.; Roote, J.; Ip, Y.T. The mesoderm determinant Snail collaborates with related zinc-finger proteins to control *Drosophila* neurogenesis. *EMBO J.* **1999**, *18*, 6426–6438.
392. Ashraf, S.I.; Ip, Y.T. The Snail protein family regulates neuroblast expression of *inscuteable* and *string*, genes involved in asymmetry and cell division in *Drosophila*. *Development* **2001**, *128*, 4757–4767.
393. Lai, S.L.; Miller, M.R.; Robinson, K.J.; Doe, C.Q. The Snail family member Worniu is continuously required in neuroblasts to prevent Elav-induced premature differentiation. *Dev. Cell* **2012**, *23*, 849–857.
394. Wei, L.; Cheng, D.; Li, D.; Meng, M.; Peng, L.; Tang, L.; Pan, M.; Xiang, Z.; Xia, Q.; Lu, C. Identification and characterization of Sox genes in the silkworm, *Bombyx mori*. *Mol. Biol. Rep.* **2011**, *38*, 3573–3584.
395. Shiraishi, H.; Kobayashi, A.; Sakamoto, Y.; Nonaka, T.; Mitsui, Y.; Aozasa, N.; Kubo, T.; Natori, S. Molecular cloning and characterization of SRAM, a novel insect Rel/Ankyrin-family protein present in nuclei. *J. Biochem.* **2000**, *127*, 1127–1134.
396. Kunte, A.S.; Matthews, K.A.; Rawson, R.B. Fatty acid auxotrophy in *Drosophila* larvae lacking SREBP. *Cell Metab.* **2006**, *3*, 439–448.
397. Meltzer, S.; Bagley, J.A.; Perez, G.L.; O'Brien, C.E.; DeVault, L.; Guo, Y.; Jan, L.Y.; Jan, Y.N. Phospholipid homeostasis regulates dendrite morphogenesis in *Drosophila* sensory neurons. *Cell Rep.* **2017**, *21*, 859–866.
398. Gujar, H.; Palli, S.R. Krüppel homolog 1 and E93 mediate Juvenile hormone regulation of metamorphosis in the common bed bug, *Cimex lectularius*. *Sci. Rep.* **2016**, *6*, 26092.
399. Emmons, R.B.; Duncan, D.; Duncan, I. Regulation of the *Drosophila* distal antennal determinant *spineless*. *Dev. Biol.* **2007**, *302*, 412–426.
400. Kwon, E.J.; Park, H.S.; Kim, Y.S.; Oh, E.J.; Nishida, Y.; Matsukage, A.; Yoo, M.A.; Yamaguchi, M.

Transcriptional regulation of the *Drosophila raf* proto-oncogene by *Drosophila* STAT during development and in immune response. *J. Biol. Chem.* **2000**, *275*, 19824–19830.
401. Gupta, L.; Molina-Cruz, A.; Kumar, S.; Rodrigues, J.; Dixit, R.; Zamora, R.E.; Barillas-Mury, C. The STAT pathway mediates late-phase immunity against *Plasmodium* in the mosquito *Anopheles gambiae*. *Cell Host Microbe* **2009**, *5*, 498–507.
402. Kanai, M.I.; Okabe, M.; Hiromi, Y. seven-up controls switching of transcription factors that specify temporal identities of *Drosophila* neuroblasts. *Dev. Cell* **2005**, *8*, 203–213.
403. Maurange, C.; Cheng, L.; Gould, A.P. Temporal transcription factors and their targets schedule the end of neural proliferation in *Drosophila*. *Cell* **2008**, *133*, 891–902.
404. Berndt, A.J.; Tang, J.C.; Ridyard, M.S.; Lian, T.; Keatings, K.; Allan, D.W. Gene regulatory mechanisms underlying the spatial and temporal regulation of target-dependent gene expression in *Drosophila* neurons. *PLoS Genet.* **2015**, *11*, e1005754.
405. Sonnenfeld, M.; Ward, M.; Nystrom, G.; Mosher, J.; Stahl, S.; Crews, S. The *Drosophila tango* gene encodes a bHLH-PAS protein that is orthologous to mammalian Arnt and controls CNS midline and tracheal development. *Development* **1997**, *124*, 4571–4582.
406. Emmons, R.B.; Duncan, D.; Estes, P.A.; Kiefel, P.; Mosher, J.T.; Sonnenfeld, M.; Ward, M.P.; Duncan, I.; Crews, S.T. The Spineless-Aristapedia and Tango bHLH-PAS proteins interact to control antennal and tarsal development in *Drosophila*. *Development* **1999**, *126*, 3937–3945.
407. Morozova, T.; Hackett, J.; Sedaghat, Y.; Sonnenfeld, M. The *Drosophila jing* gene is a downstream target in the Trachealess/Tango tracheal pathway. *Dev. Genes Evol.* **2010**, *220*, 191–206.
408. Schmidt, J.M.; Good, R.T.; Appleton, B.; Sherrard, J.; Raymant, G.C.; Bogwitz, M.R.; Martin, J.; Daborn, P.J.; Goddard, M.E.; Batterham, P., et al. Copy number variation and transposable elements feature in recent, ongoing adaptation at the *Cyp6g1* locus. *PLoS Genet.* **2010**, *6*, e1000998.
409. Jiang, Y.D.; Yuan, X.; Bai, Y.L.; Wang, G.Y.; Zhou, W.W.; Zhu, Z.R. Knockdown of *timeless* Disrupts the Circadian Behavioral Rhythms in *Laodelphax striatellus* (Hemiptera: Delphacidae). *Environ. Entomol.* **2018**, *47*, 1216–1225.
410. Steingrímsson, E.; Pignoni, F.; Liaw, G.J.; Lengyel, J.A. Dual role of the *Drosophila* pattern gene *tailless* in embryonic termini. *Science* **1991**, *254*, 418–421.
411. Mishra, A.K.; Bernardo-Garcia, F.J.; Fritsch, C.; Humberg, T.H.; Egger, B.; Sprecher, S.G. Patterning mechanisms diversify neuroepithelial domains in the *Drosophila* optic placode. *PLoS Genet.* **2018**, *14*, e1007353.
412. Czerny, T.; Halder, G.; Kloter, U.; Souabni, A.; Gehring, W.J.; Busslinger, M. *twin of eyeless*, a second Pax-6 gene of *Drosophila*, acts upstream of *eyeless* in the control of eye development. *Mol. Cell* **1999**, *3*, 297–307.
413. Castanon, I.; Von Stetina, S.; Kass, J.; Baylies, M.K. Dimerization partners determine the activity of the Twist bHLH protein during *Drosophila* mesoderm development. *Development* **2001**, *128*, 3145–3159.
414. Li, H.; Qi, Y.; Jasper, H. Ubx dynamically regulates Dpp signaling by repressing Dad expression during copper cell regeneration in the adult *Drosophila* midgut. *Dev. Biol.* **2016**, *419*, 373–381.
415. Tong, X.L.; Fu, M.Y.; Chen, P.; Chen, L.; Xiang, Z.H.; Lu, C.; Dai, F.Y. Ultrabithorax and abdominal-A specify the abdominal appendage in a dosage-dependent manner in silkworm, *Bombyx mori*. *Heredity* **2017**, *118*, 578–584.
416. Olenkina, O.M.; Egorova, K.S.; Kibanov, M.V.; Gervaziev, Y.V.; Gvozdev, V.A.; Olenina, L.V. Promoter contribution to the testis-specific expression of *Stellate* gene family in *Drosophila melanogaster*. *Gene* **2012**, *499*, 143–153.
417. Warren, J.T.; Wismar, J.; Subrahmanyam, B.; Gilbert, L.I. *Woc* (*without children*) gene control of ecdysone biosynthesis in *Drosophila melanogaster*. *Mol. Cell Endocrinol.* **2001**, *181*, 1–14.
418. Liang, H.L.; Nien, C.Y.; Liu, H.Y.; Metzstein, M.M.; Kirov, N.; Rushlow, C. The zinc-finger protein Zelda is a key activator of the early zygotic genome in *Drosophila*. *Nature* **2008**, *456*, 400–403.
419. Struffi, P.; Corado, M.; Kaplan, L.; Yu, D.; Rushlow, C.; Small, S. Combinatorial activation and concentration-dependent repression of the *Drosophila even skipped* stripe 3+7 enhancer. *Development* **2011**, *138*, 4291–4299.
420. Lai, Z.C.; Fortini, M.E.; Rubin, G.M. The embryonic expression patterns of *zfh-1* and *zfh-2*, two

Drosophila genes encoding novel zinc-finger homeodomain proteins. *Mech. Dev.* **1991**, *34*, 123–134.
421. Myllymäki, H.; Rämet, M. Transcription factor zfh1 downregulates *Drosophila* Imd pathway. *Dev. Comp. Immunol.* **2013**, *39*, 188–197.

© 2018 by the authors. Submitted for possible open access publication under the terms and conditions of the Creative Commons Attribution (CC BY) license (http://creativecommons.org/licenses/by/4.0/).
